# Choosing face: The curse of self in profile image selection

**DOI:** 10.1186/s41235-017-0058-3

**Published:** 2017-04-14

**Authors:** David White, Clare A. M. Sutherland, Amy L. Burton

**Affiliations:** 1 School of Psychology, University of New South Wales Sydney, Sydney, Australia; 2 School of Psychology, University of Western Australia, Crawley, Australia; 3 ARC Centre of Excellence in Cognition and its Disorders, Macquarie University, Sydney, NSW Australia; 4 School of Psychology, University of Sydney, Sydney, Australia

**Keywords:** Face perception, Self perception, Impression formation, Interpersonal accuracy, Online social networks, Visual communication, Photography

## Abstract

People draw automatic social inferences from photos of unfamiliar faces and these first impressions are associated with important real-world outcomes. Here we examine the effect of selecting online profile images on first impressions. We model the process of profile image selection by asking participants to indicate the likelihood that images of their own face (“self-selection”) and of an unfamiliar face (“other-selection”) would be used as profile images on key social networking sites. Across two large Internet-based studies (n = 610), in line with predictions, image selections accentuated favorable social impressions and these impressions were aligned to the social context of the networking sites. However, contrary to predictions based on people’s general expertise in self-presentation, other-selected images conferred more favorable impressions than self-selected images. We conclude that people make suboptimal choices when selecting their own profile pictures, such that self-perception places important limits on facial first impressions formed by others. These results underscore the dynamic nature of person perception in real-world contexts.

**Electronic supplementary material:**

The online version of this article (doi:10.1186/s41235-017-0058-3) contains supplementary material, which is available to authorized users.

## Significance

Selecting profile pictures is a common task in the digital age. Research suggests that choosing the right image may be critical – people’s first impressions from profile photos shape important decisions, such as choices of whom to date, befriend, or employ. Surprisingly, the process of image selection has not yet been studied directly. Here, we show that people select profile pictures that produce positive impressions on unfamiliar viewers. These impressions are tailored to fit specific networking contexts: dating images appear more attractive and professional images appear more competent. Strikingly, we show for the first time that participants select more flattering profile images when selecting pictures for other people compared with when selecting for themselves. This phenomenon has clear practical significance: should people wish to “put their best face forward,” they should ask someone else to choose it.

## Background

Key events in our professional, social, and romantic lives unfold on the Internet. Approximately one-third of employers search online for information on job candidates (Acquisti & Fong, 2015), half of British adults that are currently searching for a relationship have used online dating (YouGov, 2014), and 1.79 billion people worldwide have an active Facebook account (Facebook, 2016). As a result, we are continually forming first impressions of unfamiliar people in professional, romantic, and social contexts via social networking sites. Pictures that are chosen to represent us in these online environments—“profile images”—establish a critical link between an individual’s online and offline personas.

Profile image choices are likely to have a significant impact on the way people are perceived by others. We make inferences about an individual’s character and personality within a split second of exposure to a photograph of their face (Willis & Todorov, 2006). These impressions have been shown to predict important and diverse real-world outcomes—both online and offline—including the number of votes received by political candidates (Olivola, Funk, & Todorov, 2014), company profits generated during a CEO’s tenure (Rule & Ambady, 2008), selection as a suspect from police line-ups (Flowe & Humphries, 2011), and the popularity of an Airbnb host’s rental accommodation (Ert, Fleischer, & Magen, 2016).

Importantly, previous studies are almost exclusively based on the premise that a single image is representative of a person’s appearance. In studies of facial first impressions, participants tend to form impressions of computer-generated images or photographs captured in controlled studio conditions (e.g., expressionless, facing forwards; for a review see Todorov, Olivola, Dotsch, & Mende-Siedlecki, 2015). This procedure minimizes natural variation found in photos of faces captured outside of the laboratory. However, recent studies have emphasized the important role of this natural variability in forming social impressions. Critically, ratings of attractiveness (Jenkins, White, Van Montfort, & Burton, 2011) and character traits (Hehman, Flake, & Freeman, 2015; Todorov & Porter, 2014; cf. McCurrie et al., 2016) can vary more across different images of the same person’s face, than they do across faces of different people.

**Fig. 1:**
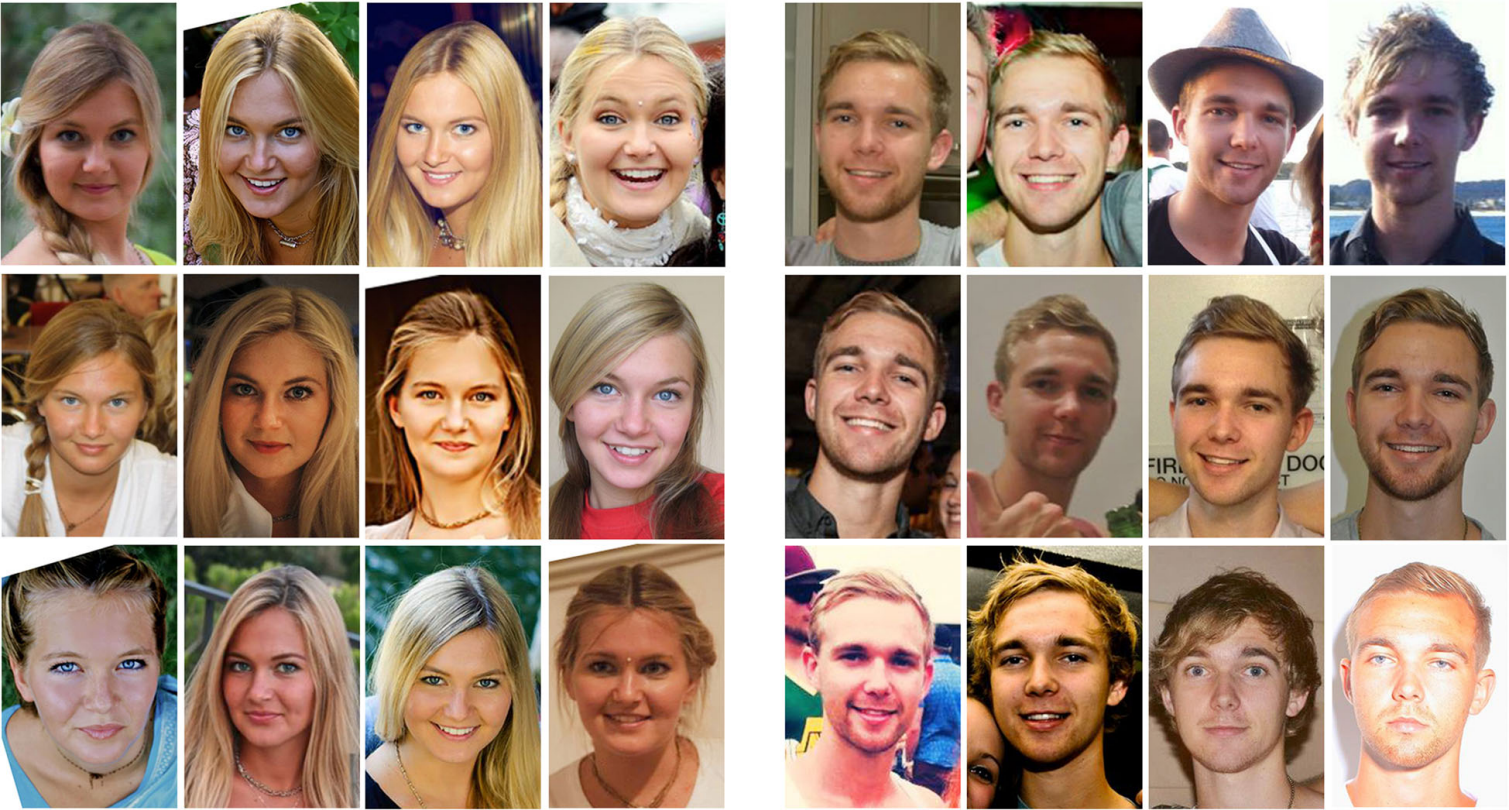
Example image sets provided by two participants in the Profile Image Dataset. Each participant selected the most and least likely image to be used in three social media contexts (see Fig. 3a), then rated the likelihood that each image would be used in each context, before rating trait impressions. They then repeated this procedure with an unfamiliar face. Images used with permission and the full Profile Image Dataset is available online in Additional file 2

In previous works, the types of images found on the Internet have been described as “ambient” photographs, as they capture dynamic aspects of faces and the environment such as expression, pose, and lighting (see Fig. 1; Jenkins et al., 2011; Sutherland et al., 2013; Vernon, Sutherland, Young, & Hartley, 2014). Importantly, influential models of social trait judgments that have been generated by ratings of studio-captured imagery (Oosterhof & Todorov, 2008) do not fully capture impressions made from ambient facial images (Sutherland et al., 2013; Todorov & Porter, 2014).

Focus on invariant aspects of facial appearance has also caused facial first impression research to overlook the importance that photograph selection has in moderating the social impact of a person’s face. However, recent work has begun to address this shortfall. In one recent study, unfamiliar viewers were able to select studio-controlled images of unfamiliar faces that accentuated traits associated with specific scenarios: for example, selecting images for a resume that accentuated impressions of competence, relative to other images of that individual (Todorov & Porter, 2014, Experiments 2 & 3). Separately, studies of impression management in online social networks have found that people report selecting images to transmit desirable impressions (Siibak, 2009) and that dating profile images tend to portray people to be more attractive than images taken in a laboratory (Hancock & Toma, 2009).

Critically, however, the process of self-selecting profile images has not been studied experimentally. Thus, while it is clear that variation in photos of the same face can modulate social impression formation (see also Jenkins et al., 2011; Wu, Sheppard, & Mitchell, 2016), it is not clear how well people exploit this variation to confer favorable impressions. This is important because perception of one’s own face is often less veridical than perception of other faces. For example, when asked to select images that represent the best likeness of themselves from photo albums, participants choose images that are less representative of their current appearance than images chosen by people with no prior familiarity (White, Burton, & Kemp, 2015). Previous studies also report systematic biases to choose images of their own face as better likenesses when they have been digitally altered to be more typical (Allen, Brady, & Tredoux, 2009), more attractive (Epley & Whitchurch, 2008; Zell & Balcetis, 2012), and more trustworthy (Verosky & Todorov, 2010); perhaps reflecting a general bias to evaluate oneself more favorably than others (Epley & Whitchurch, 2008; cf. Brown, 2012).

Given that people appear to be sensitive to variation in impressions produced by different photographs (Todorov & Porter, 2014) and are motivated to portray themselves favorably in profile images (Hancock & Toma, 2009; Siibak, 2009), we predicted that people would be able to select images of themselves to accentuate positive traits. In addition, we compared the benefit of selecting profile images of oneself to selection by an unfamiliar person. This comparison is critical in order to differentiate two, equally plausible, hypotheses: namely that self-selection may help or hinder the process of selecting favorable profile images.

On the one hand, the ability to select flattering profile images may be hindered by an impaired ability to view one’s own face accurately (e.g., White et al., 2015) and in an overly optimistic light (Epley & Whitchurch, 2008). This evidence leads to the prediction that people would select better photographs for strangers. On the other hand, this ability may be enhanced by people’s expertise in selecting flattering online photographs of themselves (Hancock & Toma, 2009) and in self-presentation more generally (e.g., Goffman, 1959; Leary & Allen, 2011; Schlenker, 2003). This reasoning leads to the opposite prediction that people would select better photographs for themselves.

We tested these predictions by examining the effect of selecting profile images on first impressions. We asked participants to indicate the likelihood that images of their own face, and of an unfamiliar face, would be used as profile images in three key social networking contexts (Facebook, dating, professional: see the “Profile Image Dataset” section). We then recruited unfamiliar viewers via the Internet to provide trait impressions of these images (see the Calibration experiment and Selection experiment sections). This approach enabled us to systematically examine the impact of photo selection on appearance-based inferences for the first time, by comparing the effect of selecting one’s own profile image (self-selection) to selection by unfamiliar others (other-selection).

## Method and results

### Profile Image Dataset

The Profile Image Dataset collected in this research consists of 12 images each of 102 students (1224 total images), downloaded from their Facebook accounts.^1^ Previous studies of photo selection have used studio-captured imagery that does not capture the full diversity of facial images shared via social media (Todorov & Porter, 2014), in terms of variations in pose, expression, and image-capture conditions across images of the same face (see Jenkins et al., 2011). Downloading photos from Facebook ensured that these were representative of variations in portrait photographs that are posted online.

In total, 114 first year undergraduate students consented to take part in the study in exchange for course credit. Participants provided 12 images in which their face: (1) took up a substantial proportion of the overall image; (2) was in clear view; (3) faced the camera; and (4) was not obscured (e.g., by sunglasses, hair, or hands). Any images not meeting these criteria or with poor resolution were rejected and the participant was asked to replace the image with another from their Facebook gallery. In total, 102 participants (51 women; mean age = 19.4 years, SD = 2.28 years) provided a full set of 12 usable images. Images were then cropped to frame the face at a fixed aspect ratio and resized to 200 × 300 pixels. Examples of images provided by two participants are shown in Fig. 1.

To capture self-selection profile image preferences, the set of 12 downloaded images were then presented on a computer monitor. Participants were asked to select which of the 12 images they were most and least likely to use as profile images for Facebook, professional (e.g., LinkedIn), and dating (e.g., Match.com) network sites. Context order was counterbalanced across subjects. The most and least likely profile images were used in the Selection experiment. After making these selections, participants then indicated their profile image preferences by rating the likelihood that they would use each of their 12 images in these contexts.

Finally, participants rated their images for five social impressions (attractiveness, trustworthiness, dominance, competence, confidence). These five ratings were made concurrently. Trustworthiness, dominance, and attractiveness were included to capture the three main dimensions of facial first impressions (Oosterhof & Todorov, 2008; Sutherland et al., 2013). Competence and confidence were included because these judgments are associated with romantic and professional success (Murphy et al., 2015; Todorov et al., 2015). Both selection likelihood and trait judgments were rated on scales from 1 (very low) to 9 (very high), and these ratings were used in the Calibration experiment.

To capture other-selection profile image preferences, participants completed an identical procedure with a set of 12 images of a randomly selected subject of the same gender that had participated in the study previously. The experimenter confirmed that the participant was unfamiliar with the person pictured in the photographs before recording their selections and instructed them to evaluate the likelihood that they would select each image if they were the person depicted. Order of self/other rating procedures was counterbalanced across participants.

### Online rating experiments

Next, we recruited new unfamiliar viewers via the Internet to rate the trait impressions produced by the Profile Image Dataset. Online ratings were collected in two experiments. First, in the Calibration experiment, we collected ratings of trait impressions to the entire image database and calculated the extent to which these first impressions were predicted by profile image preferences, provided during collection of the Profile Image Dataset. Second, in the Selection experiment, we collected ratings of trait impressions to only those images that had been explicitly selected as most/least likely to be selected as profile images. In both experiments, we examine the moderating effect of profile image preferences on first impressions; comparing the impact of participants’ preferences for images of their own face (self-selection) to preferences for images of an unfamiliar face (other-selection).

### Calibration experiment

#### Method

A total of 178 unfamiliar viewers were recruited online via the online crowdsourcing platform Amazon Mechanical Turk (M-Turk; see Buhrmester, Kwang, & Gosling, 2011) and were paid US$1. Eighteen were excluded before analysis as they reported engaging in a distracting activity during the experiment, leaving a final sample of 160 (80 women, mean age = 36.4 years; SD = 12.2 years).

Each unfamiliar viewer rated 12 different images of 12 different people (144 images presented individually in a random order). This method resulted in a pre-determined sample size of 20 raters per image that was considered sufficient to provide a stable estimate of trait impressions (see Oosterhof & Todorov, 2008). Viewers were instructed to rate how attractive, trustworthy, dominant, confident, and competent the person appeared in each image on a scale from 1 (very low) to 9 (very high). These five ratings were made on separate rating scales and scales were presented concurrently on the same screen as the photos.

#### Results

**Fig. 2:**
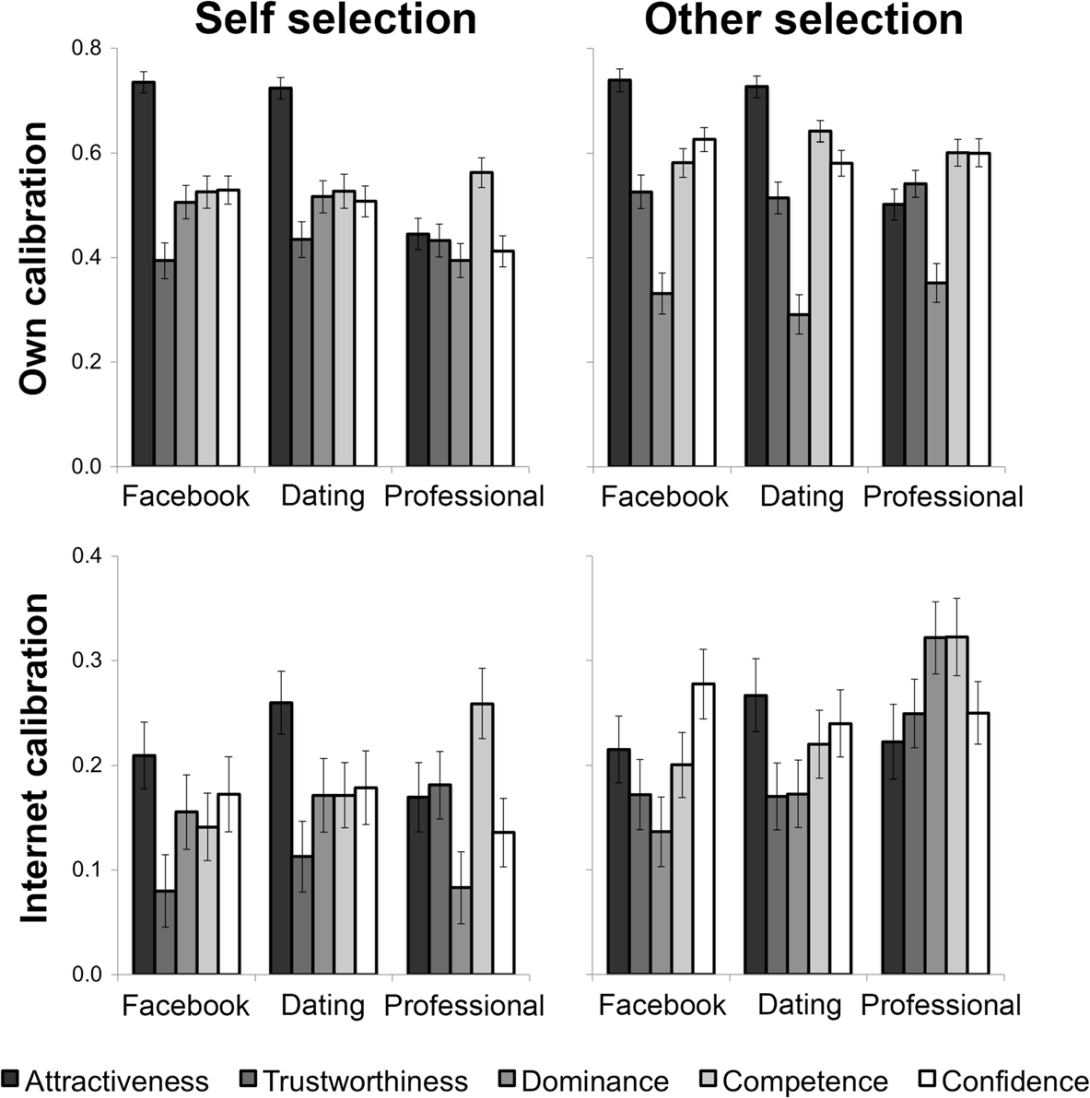
Results from the Calibration experiment. Calibration was computed separately for self-selection and other-selection as the correlation between likelihood of profile image choice and: (1) participants’ own trait impressions (*top panels*); (2) impressions of unfamiliar viewers recruited via the Internet (*bottom panels*). Higher calibration indexes participants’ ability to choose profile images that increase positive impressions. Participants’ likelihood of selecting a photograph of their own face (self-selection: *top left*) and an unfamiliar face (other-selection: *top right*) was strongly calibrated to their own impressions. However, in general, self-selections were less well calibrated to the impressions of unfamiliar viewers (*bottom left*) than were other-selections (*bottom right*). *Error bars* represent ±1 standard error

We calculated the extent to which both self-photograph and other-photograph selection likelihood ratings were calibrated with: (1) participants’ own ratings of trait impressions collected in the image collection phase (Own calibration); and (2) ratings of unfamiliar viewers trait impressions, collected via the Internet (Internet calibration).^2^ Calibration scores indexed participants’ ability to choose images that accentuated positive impressions and were calculated separately by face identity using Spearman’s rank correlation. We calculated calibration for each of the three social network contexts, to reveal which traits were most accentuated by profile image selection in each context, and analyzed these data separately for own and Internet ratings. Results of this analysis are shown in Fig. 2.

Own and Internet calibration scores were analyzed by mixed ANOVA with between-subject factor of Selection Type (self, other) and within-subject factors Context (Facebook, dating, professional) and Trait (attractiveness, trustworthiness, dominance, competence, confidence). For own calibration, the main effect of Selection Type was non-significant, F (1,202) = 1.48, *p* = 0.25, η_p_^2^ = 0.007, with high average calibration between image selection and positive social impressions for both self-selected (M = 0.509; SD = 0.319) and other-selected photographs (M = 0.543; SD = 0.317). For Internet calibration, the main effect of Selection Type was significant, F (1,202) = 4.12, *p* = 0.044, η_p_^2^ = 0.020. Critically, there was greater calibration between image selection and positive social impressions for other-selected (M = 0.227; SD = 0.340) compared to self-selected photographs (M = 0.165; SD = 0.344).

In both own and Internet calibration analysis, the interaction between Context and Selection Type was significant (Own: F [2, 404] = 4.16, *p* = 0.016, η_p_^2^ = 0.020; Internet: F [2, 404] = 4.26, *p* = 0.015, η_p_^2^ = 0.021), reflective of higher calibration for other-selections compared to self-selections in professional (Own: F [1, 202] = 5.73, p = 0.018, η_p_^2^ = 0.028; Internet: F [1, 202] = 11.16, *p* < 0.000, η_p_^2^ = 0.052) but not Facebook or dating contexts (all Fs < 1). In general, interactions revealed that traits were aligned to network contexts, such that attractiveness tended to calibrate most with social and dating networks and competence and trustworthiness to professional networks (see Additional file 1 for full details of this analysis).

#### Discussion

Consistent with predictions based on studies of self-presentation (e.g., Hancock & Toma, 2009; Siibak, 2009), the pattern of results observed in the Calibration experiment lends broad support to the notion that people select images of themselves to accentuate positive trait impressions and that these selections are fitted to specific social networking contexts (cf. Leary & Allen, 2011). Strikingly, however, the profile image preferences indicated in other-selections were more calibrated to impressions formed by unfamiliar viewers than self-selections. This result is contrary to the prediction based on self-presentation literature, that participants would select more flattering images of themselves than of other people.

Notably, the cost of self-selection applied only to professional profile image selections, raising the possibility that costs of self-selection were specific to this network context. Therefore, in a second experiment, we again examined effects of self-selection on first impressions, but using a more direct test: comparing trait judgments to images that had been explicitly chosen as most and least likely to be used as profile images for different network contexts (see “Profile Image Dataset” method).

In the Calibration experiment, unfamiliar viewers also rated 12 images of a single individual, making it likely that this diluted their first impressions. Further, these viewers made multiple trait judgments to a single photo, which may increase overlap in these judgments (Rhodes, 2006). We addressed these potential concerns in the Selection experiment, by now presenting unfamiliar viewers with only two images of each participant (most/least likely profile image choice) and asking viewers to rate these images for a single trait impression.

### Selection experiment

#### Method

A total of 482 new unfamiliar viewers were recruited online via M-Turk and were paid US$1. Data from 50 viewers were excluded from the analysis because they did not pass the quality criteria used in the previous experiment, leaving a final sample of 432 (273 women), with an average age of 36.4 years (SD = 11.6 years).

**Fig. 3:**
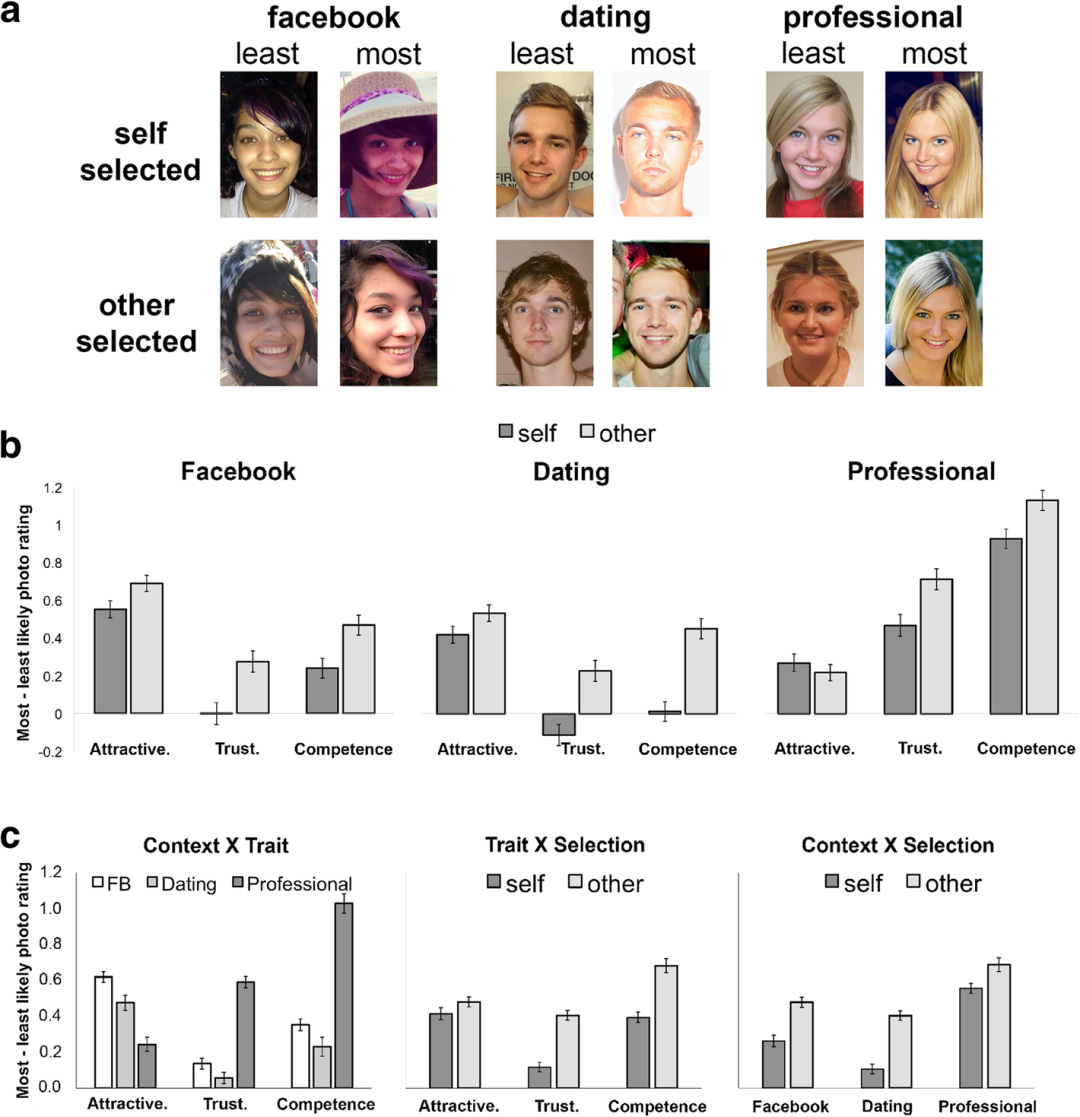
**a** Examples of most and least likely image selections used in the Selection experiment. Images are used with permission and the full set of experimental materials are available online in Additional file 5. **b** Mean difference between trait impression ratings to photographs chosen as most and least likely profile pictures for each of three contexts. Positive values signify higher trait ratings for images selected as “most likely” profile images, again revealing more positive first impressions for images that were selected by an unfamiliar other (*light gray*) when compared to self-selections (*dark gray*). **c** Significant two-way interactions (see text for details of analysis). All *error bars* denote ±1 standard error

In this experiment, we focused on impressions of attractiveness, trustworthiness, and competence. Viewers rated images that had been selected by participants in the Profile Image Dataset as being most and least likely to be used in each social network context. This procedure resulted in 12 images of each pictured identity (3 contexts × self/other selected × least/most likely; Fig. 3a). To balance the design of the Selection experiment we randomly selected a subset of 96 pictured identities from the Profile Image Dataset. A total of 1152 images were divided into 12 counterbalanced versions of the experiment. This method resulted in a sample size of 36 viewers per counterbalanced version. Each viewer rated 192 images on a single trait (attractiveness, trustworthiness, competence), with each pictured identity appearing twice (most and least likely images from one combination of Context/Selection Type). The experimental design ensured that assignment of pictured identities to conditions was counterbalanced across viewers.

#### Results

Difference scores were calculated separately for each viewer in the Selection experiment by subtracting their mean trait ratings to “least likely” images from ratings to “most likely” images. This provided a measure of the effect of image selection on facial first impressions at the level of the viewer. These data were analyzed by using a mixed-factor ANOVA with between-subject factor of Trait (attractiveness/trustworthiness/competence) and within-subject factors of Selection Type (self/other) and Context (Facebook/dating/professional). Mean difference scores for each condition are shown in Fig. 3b.

This analysis revealed a significant main effect of Selection Type, F (2, 429) = 77.2; *p* < 0.001, η_p_^2^ = 0.152, with other-selections again enhancing trait impressions more than self-selections. The main effect of Context was also significant, F (2, 858) = 78.7, *p* < 0.001, η_p_^2^ = 0.155, with image selection having the greatest effect on trait judgments in professional network (M = 0.621; SD = 0.787) compared with Facebook (M = 0.370; SD = 0.657) and dating contexts (M = 0.255; SD = 0.587).

Main effects were qualified by three two-way interactions. First, the interaction between Context and Trait was significant (see Fig. 3c [left]: F [4, 858] = 73.8; *p* < 0.001 η_p_^2^ = 0.256), indicating that different traits were accentuated in different online contexts. Specifically, selections for Facebook (M = 0.619; SD = 0.355) and dating (M = 0.475; SD = 0.366) accentuated ratings of attractiveness more than professional networking selections (M = 0.246; SD = 0.380). Selections for professional networking contexts conferred significantly more benefit to trustworthiness (M = 0.590; SD = 0.648) and competence (M = 1.029; SD = 0.638) relative to selections for Facebook (Trustworthiness: M = 0.137; SD = 0.470, Competence: M = 0.353; SD = 0.503) and Dating (Trustworthiness: M = 0.058; SD = 0.372, Competence: M = 0.232; SD = 0.391).

Second, the interaction between Selection Type and Trait was significant (see Fig. 3c [middle]: F [4, 858] = 9.18; *p* < 0.001; η_p_^2^ = 0.041). The benefit of other-selection over self-selection was carried by other-selections conferring more positive impressions for trustworthiness, F (1, 429) = 46.2; *p* < 0.001; η_p_^2^ = 0.103, and competence, F (1, 429) = 46.8; *p* < 0.001; η_p_^2^ = 0.104. Interestingly, other-selections did not confer a significant benefit for attractiveness impressions, F (1, 429) = 2.47; *p* > 0.05; η_p_^2^ = 0.012. Third, the interaction between Selection Type and Context was significant (see Fig. 3c [right]: F [4, 858] = 9.18; *p* < 0.001; η_p_^2^ = 0.041). Other-selections produced more positive effects on trait impressions in comparison to self-selection across all contexts, but to differing degrees (Facebook: F [1, 429] = 27.6; *p* < 0.000; η_p_^2^ = 0.063; dating: F [1, 429] = 53.1; *p* < 0.001; η_p_^2^ = 0.112; professional: F [1, 429] = 10.5; *p* = 0.001; η_p_^2^ = 0.024).

#### Discussion

Results of the Selection experiment replicated the main findings of the previous experiment. First, profile image selection accentuated positive first impressions and these impressions were matched to specific network contexts. This confirms that people are aware of the different impressions that different images confer and adjust their choices to fit the particular context. Second, and more surprisingly, self-selected profile images conferred less favorable impressions when compared to other-selected images. Whereas this effect was limited to professional networking contexts in the Calibration experiment, using a more sensitive test in the Selection experiment, we observed the effect across all networking contexts.

## General discussion

This paper reports the first systematic test of people’s profile image selection behavior. Strikingly, we found that people selected images of themselves that cast less favorable first impressions than images selected by strangers. At face value, this result appears to run contrary to a vast literature showing that people portray themselves more positively than other people. Self-enhancement is a pervasive human tendency in a variety of social contexts (e.g., Goffman, 1959; Schlenker, 2003), including social networking sites (see Hancock & Toma, 2009; Siibak, 2009). Interestingly, pioneering work by Erving Goffman conceptualized self-presentation as a process of projecting deliberately choreographed “face” to others (Goffman, 1955) and a large literature shows that people manage their appearance to improve likelihood of desirable outcomes.

Given this apparent expertise in showing face, it might be expected that people would also be experts in choosing face: they would be more adept at selecting favorable facial images of themselves than they would be at selecting favorable facial images of unfamiliar people. However, our results clearly argue against any such self-expertise.

Although our results are surprising in the context of self-enhancement research, they may be related to the finding that people tend to perceive themselves more positively than other people. For example, it has been shown that people evaluate images of one’s own face as more trustworthy than unfamiliar faces (Verosky & Todorov, 2010). Importantly, the task faced when selecting profile images is to discriminate between images of your own face. The existence of positivity biases is therefore unlikely to improve a person’s ability to make these selections, if such biases are independent of discrimination (cf. Macmillan & Creelman, 2004). One apparently plausible account of our findings is that, somewhat paradoxically, these self-enhancing biases in perception may in fact interfere with a person’s ability to discriminate between images when selecting one to portray a positive impression.

Although plausible, this account of self-selection costs is inconsistent with the fact that costs were specific to certain trait impressions. In the “Selection experiment,” although we observed overall costs within each social network context, costs were nevertheless specific to impressions of trustworthiness and competence and were not observed for attractiveness. Previous studies have shown that people perceive their own face to be both more trustworthy (Verosky & Todorov, 2010) and more attractive than other people’s faces (Epley & Whitchurch, 2008; Zell & Balcetis, 2012). Explanations of self-selection costs in terms of self-enhancing biases are not able to account for the fact that we observed costs in one trait evaluation but not the other. This in turn suggests that the mechanisms responsible for self-enhancing biases, and the cost of self-selection reported here, are relatively independent.

Given that this is the first report of self-selection costs in profile image choice, future research is necessary to elucidate the precise mechanisms underlying these costs. In particular, it will be important to examine the contribution of familiarity more closely. Recent work shows similar self-selection costs when choosing images that are representative of our current appearance: people choose images of themselves that are less representative than images chosen by unfamiliar viewers after brief familiarization (White et al., 2015). This shows that difficulties in selecting images of our own face are not specific to socially motivated tasks. Interestingly, very recent evidence suggests that memory for specific images of familiar faces may be impaired relative to unfamiliar faces (Armann, Jenkins, & Burton, 2016); raising the possibility that familiarity for any face—not only our own face—causes difficulty in discriminating between different images of that face. Future studies designed to test this possibility can help to separate contributions of visual familiarity from the broader cognitive system of self-representation (see Devue & Brédart, 2011).

Notwithstanding a large cost of self-selection, we found that first impressions were substantially enhanced by profile image selection and these selections were tailored to social networking contexts. Overall, participants were aware of the impressions made by different images of their face and made profile image choices accordingly, fitting facial first impressions to the social context of the audience. This extends recent work showing that people can detect subtle differences in impressions made by different photos of the same unfamiliar face, both when photos are captured in controlled studio conditions (Todorov & Porter, 2014) and in ambient environments (Jenkins et al., 2011). In parallel, computer scientists have made impressive progress in developing automated methods for predicting human’s first impressions from ambient facial imagery. Using deep neural networks trained on human’s ratings of first impressions, McCurrie et al. (2016) were able to predict facial first impressions from face images relatively accurately (cf. Vernon et al., 2014). In future work, it may be useful to compare human profile selection choices to these computational benchmarks.

More broadly, our results have implications for self-presentation in modern society. Recent data show that 1.8 billion images are uploaded every day to popular social networking sites (KPCB, 2014), leading to a multitude of new opportunities for self-monitoring behavior (see also Hancock & Toma, 2009; Siibak, 2009; Van Dijck, 2008; Walther, 1996). Self-selection of images is a multi-staged process: taking “selfies” (see Re, Wang, He, & Rule, 2016); deleting images from digital cameras; selecting images to upload to social media; “untagging” images on Facebook (see Lang & Barton, 2015). In this context, an important limitation of the present study is that images were initially downloaded from Facebook. Therefore, selection behavior reported in this paper may represent the final stage in a hierarchy of selection filters that combine to determine a person’s online appearance. Nevertheless, given the robust cost of self-selection we observe here, it is likely that this effect serves to limit positive facial impressions at multiple levels in this hierarchy, thereby curtailing people’s ability to “put their best face forward.”

## Conclusions

Given the diverse opportunities for self-monitoring via digital media, understanding the dynamics of selection behavior will be important in developing models of facial first impressions that are relevant to real-world social networking contexts. We propose that image selection tasks can provide a lens through which to understand processes that modulate the signaling and receiving of these impressions in daily life—from current impression management goals to inherent perceptual abilities. For now, it is clear that the facial first impressions we transmit to unfamiliar people—via online social networks—are constrained by how we perceive ourselves. Our results also impart practical wisdom: when it comes to choosing the best version of ourselves, it may be wise to let other people choose for us.

## Additional files

Additional file 1: Full description of analysis in the Calibration experiment. (PDF 166 KB)

Additional file 2: Images used in the Calibration experiment. (PDF 16.7 MB)

Additional file 3: Raw rating data from Calibration experiment. (XLSX 107 KB)

Additional file 4: Spearman's rho scores from Calibration experiment. (XLSX 125 KB)

Additional file 5: Images used in the Selection experiment. (PDF 17.0 MB)

Additional file 6: Rating data from the Selection experiment (by viewer). (XLSX 87.4 KB)

Additional file 7: Rating data from the Selection experiment (by image). (XLSX 528 KB)

## Footnotes

1 All images and accompanying rating data are available in Additional files. Participants have consented to the use of their images in future research. To protect participants’ privacy, the mapping between images and rating data has been withheld. Should researchers require this information, the full Profile Image Dataset is available from the authors on request.

2 Because average ratings of M-Turk raters were used to compute calibration, we checked the stability of these ratings across subjects using Cronbach’s Alpha. This analysis confirmed high levels of reliability for all impressions (Attractiveness = 0.893, Trustworthiness = 0.821, Dominance = 0.721, Competence = 0.756, Confidence = 0.785).
