# Correction to: Choosing face: The curse of self in profile image selection

**DOI:** 10.1186/s41235-021-00320-2

**Published:** 2021-08-13

**Authors:** David White, Clare A. M. Sutherland, Amy L. Burton

**Affiliations:** 1 School of Psychology, University of New South Wales Sydney, Sydney, Australia; 2 School of Psychology, University of Western Australia, Crawley, Australia; 3 ARC Centre of Excellence in Cognition and Its Disorders, Macquarie University, Sydney, NSW Australia; 4 School of Psychology, University of Sydney, Sydney, Australia

## Correction to: Cognitive Research: Principles and Implications (2017) 2:23 10.1186/s41235-017-0058-3

When carrying out further analysis of the rating and image database associated with our publication (White et al., 2017), we detected a data processing error. After a detailed investigation and re-analysis of the dataset, we found that this error affected participant rating data matrices in the ‘Internet calibration’ analysis presented in the bottom two panels of Fig. 2 in the main manuscript.

The corrected summary data are shown in Figure E1, and the corrected text associated with statistical tests performed on these data is reported in this analysis below. We have also updated the full analysis of these data in Additional file 1.

This error does not change any of the main results in the study and nor does it affect our conclusions. The main effects reported in the manuscript are strengthened relative to the published report (published ηp^2^ for main effect of self/other selection = 0.020, corrected ηp^2^ = 0.026). There are some changes to the qualitative pattern of the interaction between trait and profile picture context type (facebook, dating, professional). In the published paper, these interactions were reported in Supplemental Material (Additional file 1) as they were of marginal interest and unrelated to the main result reported in the paper.

Although the main pattern of results is unchanged, this error may impact users of the image and rating dataset that we made available for future work. We are aware of two research labs that have used this dataset and have contacted them advising of the situation. Additional files 3 and 4 that contained affected rating data have also been replaced with the corrected versions on the url associated with the original publication. We hope that researchers will continue to use this corrected version to explore the relationship between image variation and facial first impressions in their work.

**Fig. E1:**
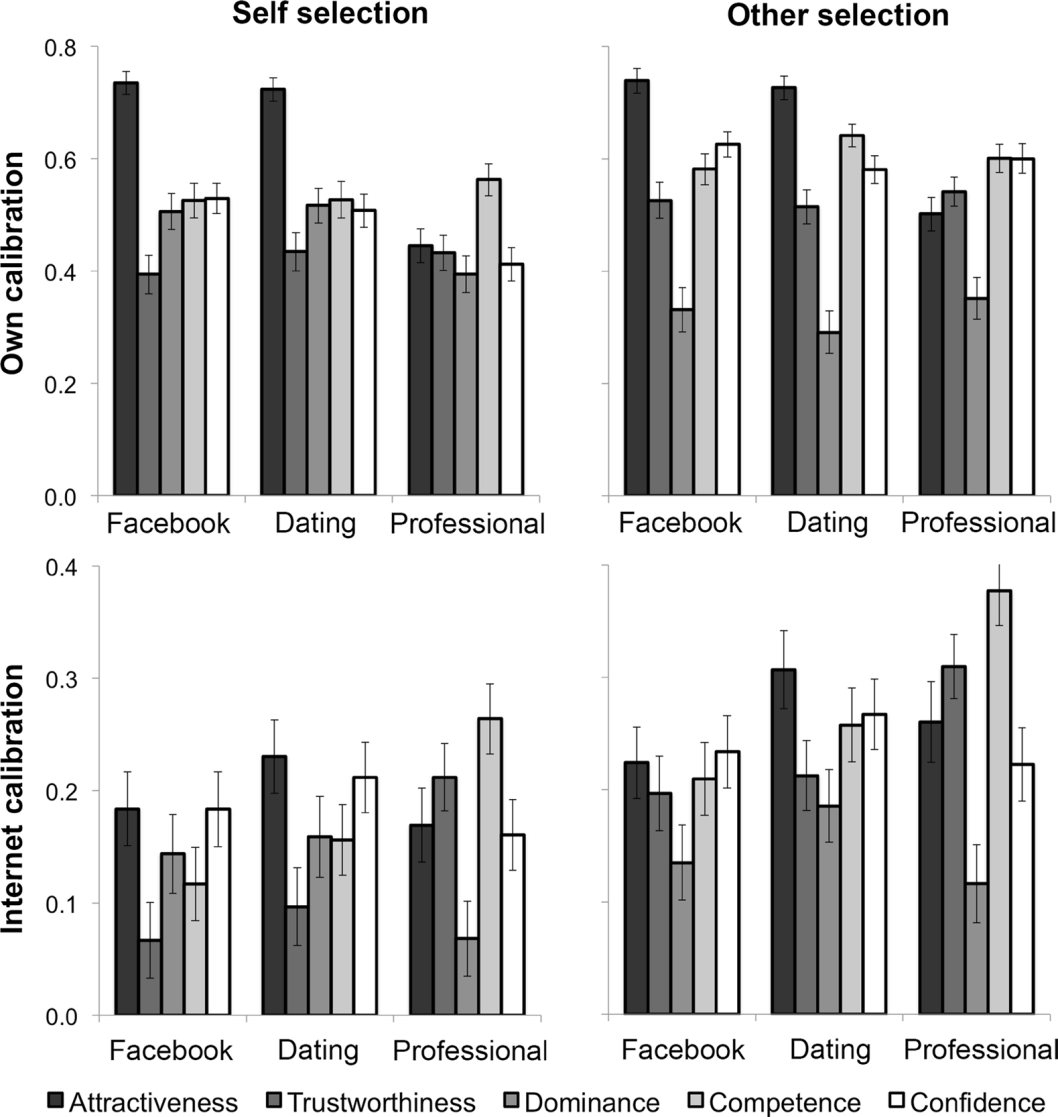
Corrected results from the Calibration experiment. The data in the upper panels remain unchanged from the original publication, but the data in lower panels has changed subsequent to correction

**Corrected text for paragraphs 2 and 3 of the Calibration Experiment, Results section (corrected stats in bold italics)**:

Own and Internet calibration scores were analyzed separately by mixed ANOVAs with between-subject factor of Selection Type (self, other) and within-subject factors Context (Facebook, dating, professional) and Trait (attractiveness, trustworthiness, dominance, competence, confidence). For own calibration, the main effect of Selection Type was non-significant, F (1,202) = 1.48, *p* = 0.25, η_p_^2^ = 0.007, with high average calibration between image selection and positive social impressions for both self-selected (M = 0.509; SD = 0.319) and other-selected photographs (M = 0.543; SD = 0.317). For Internet calibration, the main effect of Selection Type was significant, ***F (1,202) = 5.50, p = 0.02, ηp 2 = 0.026***. Critically, there was greater calibration between image selection and positive social impressions for other-selected (***M = 0.234; SD = 0.327***) compared to self-selected photographs (***M = 0.161; SD = 0.336***)*.*

Interaction between Context and Selection Type was significant for own rating calibration, F [2, 404] = 4.16, *p* = 0.016, η_p_^2^ = 0.020, reflective of higher calibration for other-selections compared to self-selections in professional (F [1, 202] = 5.73, p = 0.018, η_p_^2^ = 0.028) but not Facebook or dating contexts (all Fs < 1). However, neither of these interactions were significant for Internet calibration, meaning that the benefit of other people selecting profile images for internet ratings was consistent across contexts and traits (***both interactions Fs < 1***).

In general, interactions revealed that traits were aligned to network contexts, such that attractiveness tended to calibrate most with social and dating networks and competence and trustworthiness to professional networks (see Additional file 1 for full details of this analysis).

## Supplementary Information

**Additional file 1**. Full description of analysis in the Calibration experiment.

**Additional file 3**. Raw rating data from Calibration experiment.

**Additional file 4**. Spearman's rho scores from Calibration experiment.
