# Supplementary material for: Choosing face: The curse of self in profile image selection
Source: Cogn Res Princ Implic. 2017 Apr 14;2:23. doi: 10.1186/s41235-017-0058-3 (PMC5391387; doi:10.1186/s41235-017-0058-3)
Supplement: Supplementary file 1 — Full description of analysis in the Calibration experiment. (PDF 166 KB) [file 41235_2017_58_MOESM1_ESM.pdf]

The curse of self in profile image selection

Additional File 1: Calibration Experiment Analysis

David White*, Clare A. M. Sutherland, Amy L. Burton

Corresponding author: david.white@unsw.edu.au

Calibration data were computed as Spearman's Rho coefficients between selection likelihood ratings (Facebook, dating, professional) and trait ratings (attractiveness, dominance, trustworthiness, competence, confidence). These calibration scores were computed between the likelihood ratings and selectors own trait ratings, and also between likelihood ratings and trait ratings by a group of unfamiliar viewers recruited via the Internet. These data are summarized in Figure 2, and provided in full in Additional File 3. Calibration scores were analysed by separate three-way mixed factor ANOVA with between-subject factor of Selection Type (self, other) and within-subject factors Context (Facebook, dating, professional) and Trait (attractiveness, dominance, trustworthiness, competence, confidence).

Own ratings

For own ratings, the main effect of Selection Type was non-significant, F (1, 202) = 1.48, p = 0.225, $\eta_p^2 = 0.007$. Main effects of Trait, F (4, 808) = 45.5, p = 0.003, $\eta_p^2 = 0.184$, and Context, F (2, 404) = 22.3, p = 0.011, $\eta_p^2 = 0.099$, were highly significant. There was a significant two-way interaction between Trait and Selection Type, F (4, 808) = 13.9, p < 0.000, $\eta_p^2 = 0.065$. Analysis of Simple Main Effects revealed that this interaction was driven by: (i) significantly lower calibration of self-selection with trustworthiness ratings, F (1, 202) = 9.75, p = 0.002, and competence ratings, F (1, 202) = 5.49, p = 0.020, compared to other-selection calibrations; (ii) *higher* calibration of self-selection with selector's own dominance ratings, F (1, 202) = 10.6, p = 0.001; (iii) a non-significant difference between self and other selection calibration for attractiveness ratings, F (1, 202) = 0.68, p = 0.411.

The interaction between Context and Selection Type, F (2, 404) = 4.16, p = 0.016, $\eta_p^2 = 0.020$, was also significant, reflective of a higher calibration between selection likelihood and selectors own trait ratings for other-selections in professional, F (1, 202) = 5.73, p = 0.018, $\eta_p^2 = 0.028$, but not Facebook, F (1, 202) = 0.413, p = 0. 521, $\eta_p^2 = 0.002$, or dating contexts, F (1, 202) = 0.035, p = 0.852, $\eta_p^2 < 0.000$.

A significant two-way interaction between Trait and Context was also observed, F (8, 1616) = 22.3, $p = 0.011$, $\eta_p^2 = 0.099$, but this was qualified by a significant three-way interaction, F (8, 1616) = 3.73, $p < 0.000$, $\eta_p^2 = 0.018$. In light of the three-way interaction, we analysed Trait and Context interaction separately for self and other selection. Calibration of self-selection to Trait varied as a function of selection Context for attractiveness, F (2, 1010) = 54.9, $p < 0.001$, $\eta_p^2 = 0.10$, dominance, F (2, 1010) = 9.32, $p < 0.001$, $\eta_p^2 = 0.02$, and confidence, F (2, 1010) = 7.89, $p < 0.001$, $\eta_p^2 = 0.02$ (with non-significant main effects of trustworthiness, F (2, 1010) = 1.06, $p > 0.05$, and competence F (2, 1010) = 0.90, $p > 0.05$). For other-selection, calibration between varied as a function of selection Context only for attractiveness ratings, F (2, 1010) = 55.1, $p < 0.001$, $\eta_p^2 = 0.10$. (Non-significant simple main effects of trustworthiness: F (2, 1010) = 0.57, $p > 0.05$; dominance: F (2, 1010) = 2.90, $p > 0.05$; competence: $F(2, 1010) = 2.89$, $p >0.05$; confidence: F (2, 1010) = 1.52, $p > 0.05$).

Internet ratings

For Internet ratings, the main effect of Selection Type was significant, F (1, 202) = 4.12, $p = 0.044$, $\eta_p^2 = 0.020$. Main effects of Trait, F (4, 808) = 3.96, $p = 0.003$, $\eta_p^2 = 0.019$, and Context, F (2, 404) = 4.54, $p = 0.011$, $\eta_p^2 = 0.022$, were also significant. The interaction between Context and Selection Type was significant, F (2, 404) = 4.26, $p = 0.015$, $\eta_p^2 = 0.021$, reflecting an overall benefit of self-selection for professional networking context, F (1, 202) = 11.16, $p < 0.000$, $\eta_p^2 = 0.052$, but not in compared to other contexts [Facebook: F (2, 202) = 1.73, $p = 0.190$, $\eta_p^2 = 0.008$; Dating: F (1, 202) = 0.687, $p = 0.408$, $\eta_p^2 = 0.003$].

The interaction between Trait and Selection Type was non-significant, F (4,808) = 0.74, $p = 0.562$, $\eta_p^2 = 0.004$. The interaction between Trait and Context was significant, F (8, 1616) = 10.98, $p < 0.000$, $\eta_p^2 = 0.052$, but this was qualified by a significant three-way interaction, F (8, 1616) = 6.21, $p < 0.000$, $\eta_p^2 = 0.030$.

Given the three-way interaction, we analysed Trait and Context interactions separately for self and other selection calibration. This interaction was significant for both self, F (4, 808) = 10.18, $p < 0.000$, $\eta_p^2 = 0.09$, and other selection, F (4, 808) = 7.54, $p < 0.000$, $\eta_p^2 = 0.07$.

However, qualitatively different patterns emerged. For self-selection, Simple Main Effects of Context were significant for attractiveness, $F(2, 1010) = 4.76$, $p < 0.01$, $\eta_p^2 = 0.01$, trustworthiness, $F(2, 1010) = 6.20$, $p < 0.01$, $\eta_p^2 = 0.01$, dominance, $F(2, 1010) = 5.14$, $p < 0.01$, $\eta_p^2 = 0.01$, and competence, $F(2, 1010) = 8.69$, $p < 0.001$, $\eta_p^2 = 0.02$, (but non-significant for confidence: $F(2, 1010) = 1.25$, $p > 0.05$, $\eta_p^2 < 0.00$). For other-selection, Simple Main Effects of Context were significant for trustworthiness, $F(2, 1010) = 7.18$, $p < 0.001$, $\eta_p^2 = 0.01$, dominance, $F(2, 1010) = 21.31$, $p < 0.001$, $\eta_p^2 = 0.04$, and competence, $F(2, 1010) = 9.48$, $p < 0.001$, $\eta_p^2 = 0.02$, (but non-significant for attractiveness, $F(2, 1010) = 1.72$, $p > 0.05$, $\eta_p^2 < 0.00$, and confidence ratings, $F(2, 1010) = 0.84$, $p > 0.05$, $\eta_p^2 < 0.00$).
