# Supplementary material for: Choosing face: The curse of self in profile image selection
Source: Cogn Res Princ Implic. 2017 Apr 14;2:23. doi: 10.1186/s41235-017-0058-3 (PMC5391387; doi:10.1186/s41235-017-0058-3)

The curse of self in profile image selection

D. White[1], C. A. M. Sutherland[2], A. L. Burton[3]

[1]UNSW Australia; [2]University of Western Australia; [3]University of Sydney

<u>Additional File 2:</u>
<u>Stimuli used in *Calibration Experiment*</u>

Twelve images of each participant who consented for their images to be published are presented in rows. Images and identities are presented in random order.

To ensure anonymity of participant data, we do not provide participant numbers associated with images here.

A full version of the Profile Image Database, enabling mapping of images to item rating data are available for use in academic research by contacting the lead author:
david.white@unsw.edu.au / davidwhitephd@gmail.com

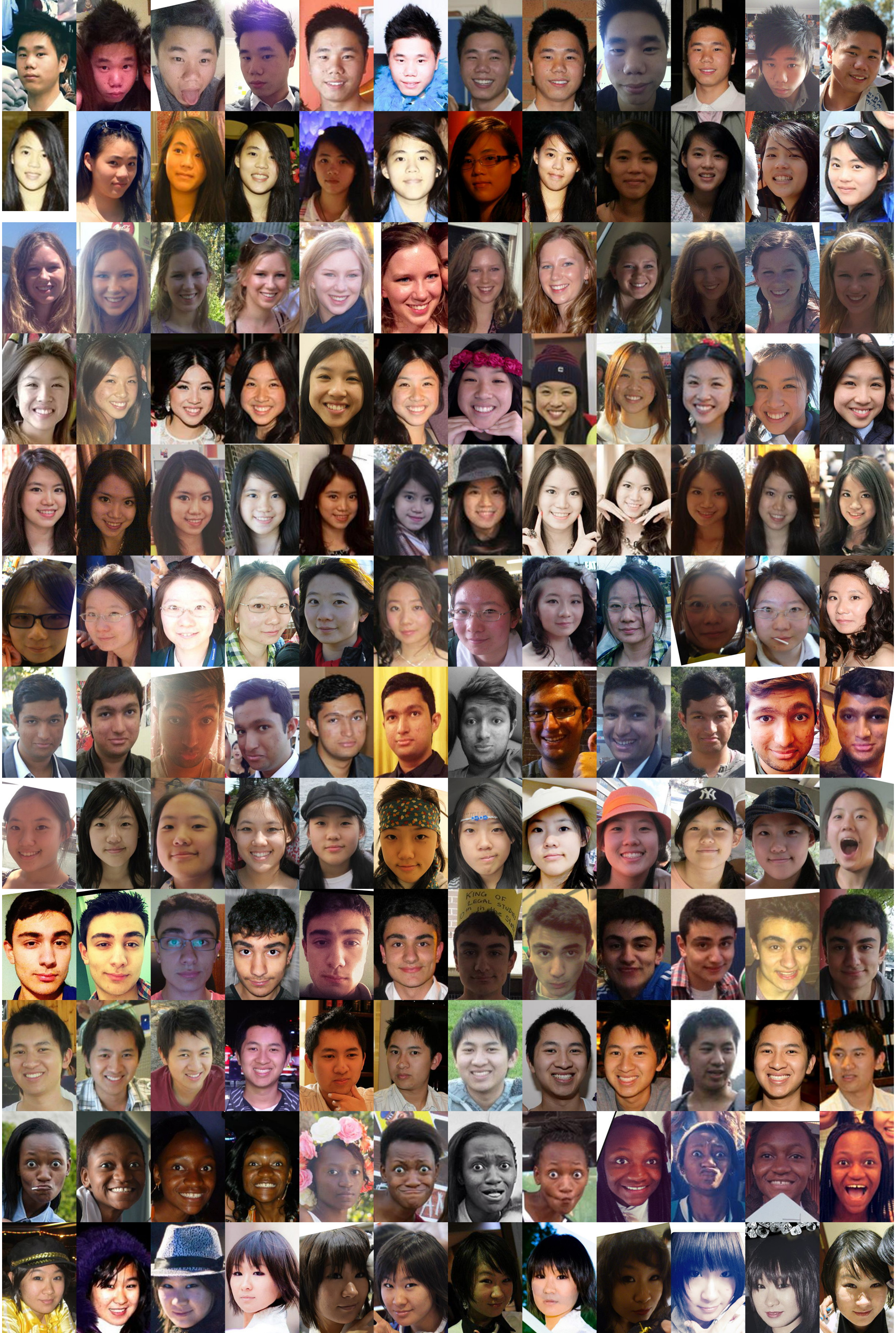

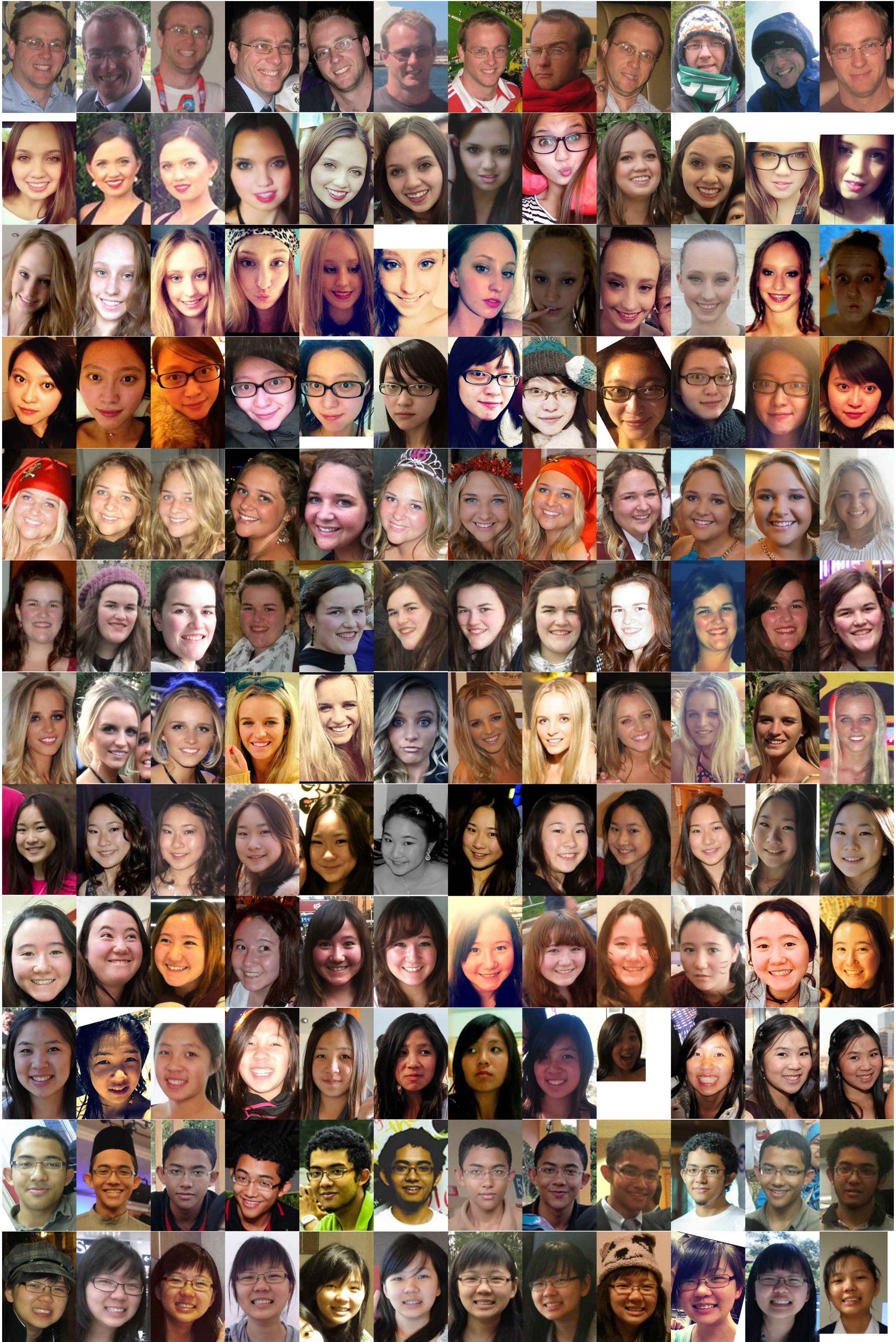

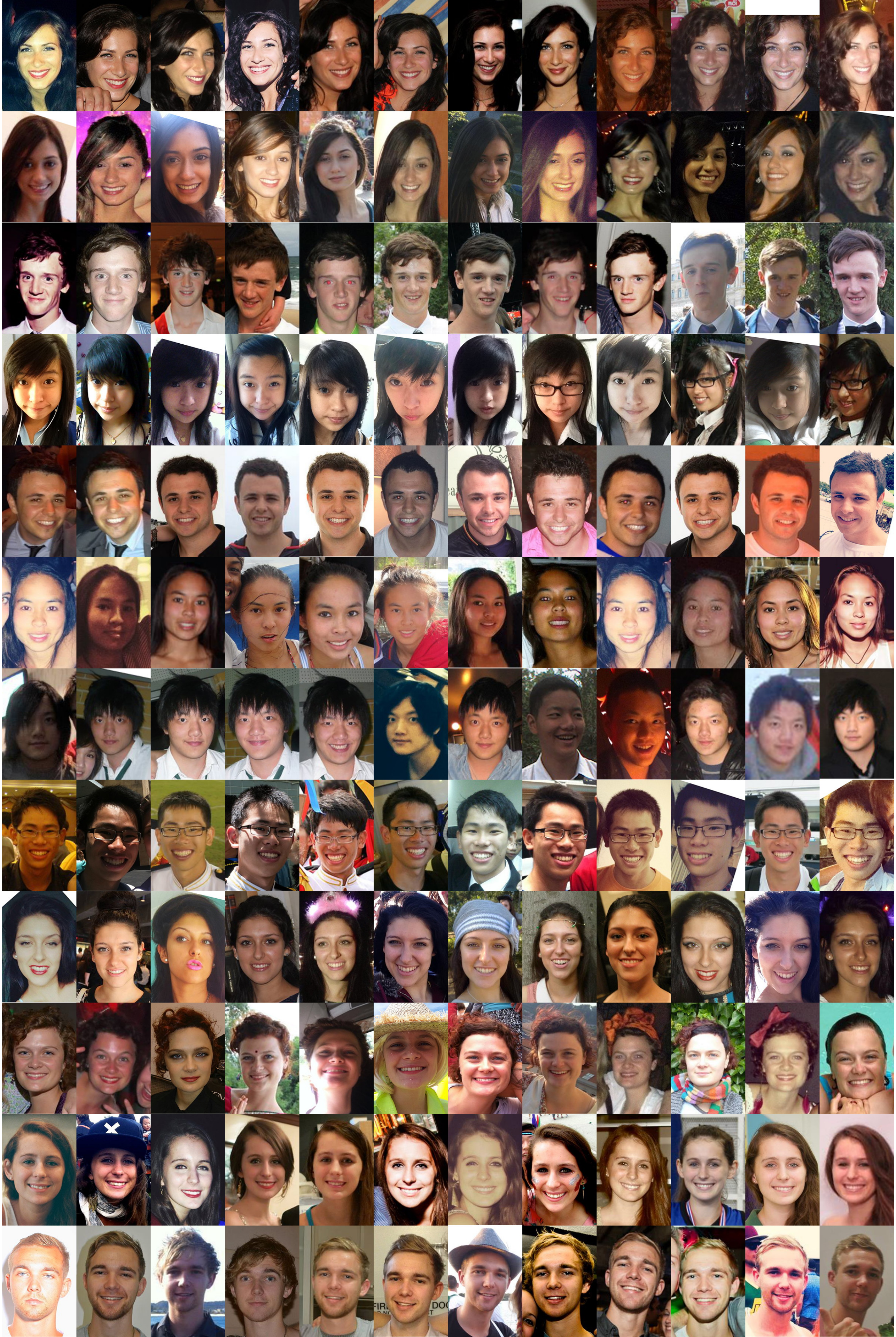

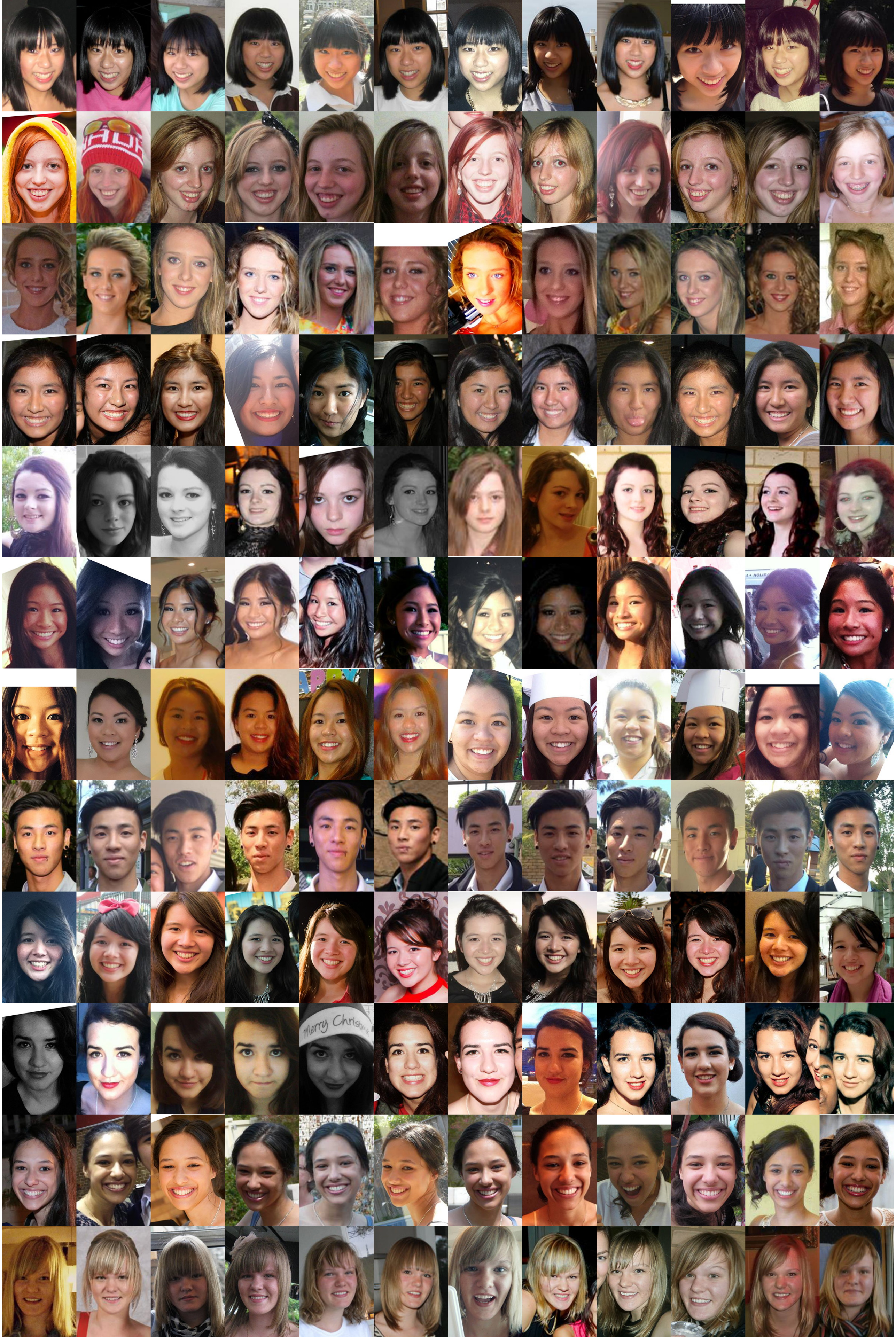

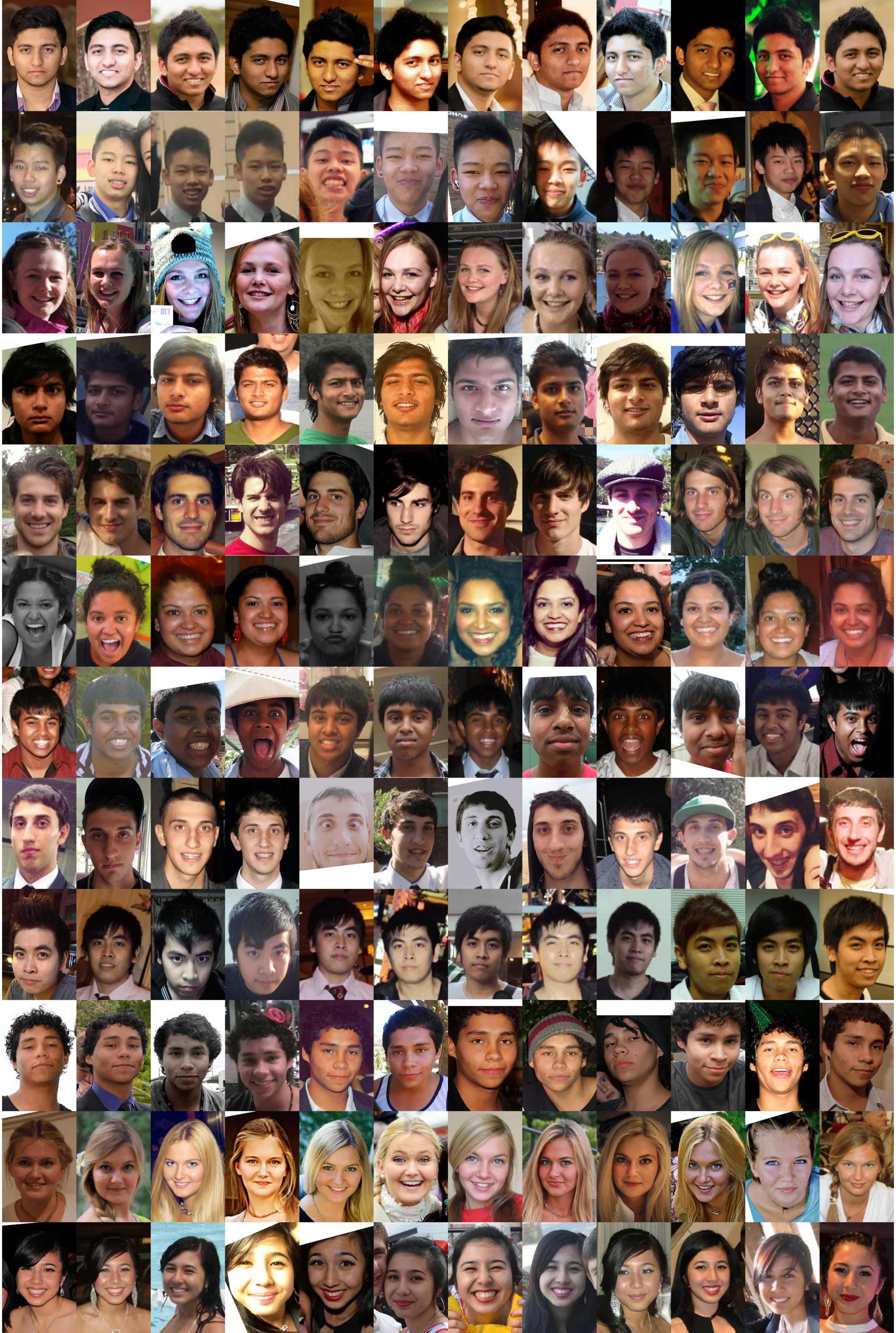

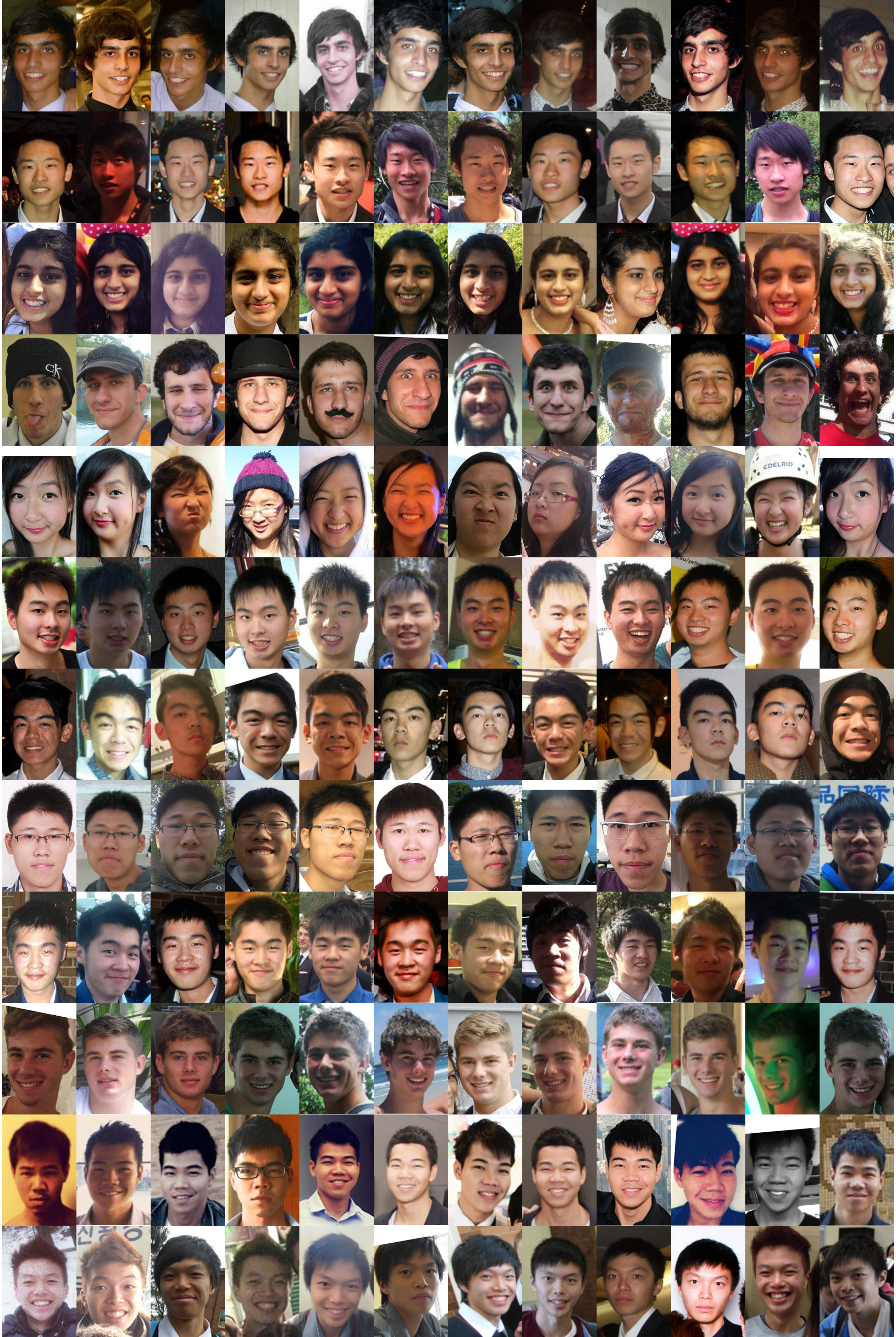
EDELRID

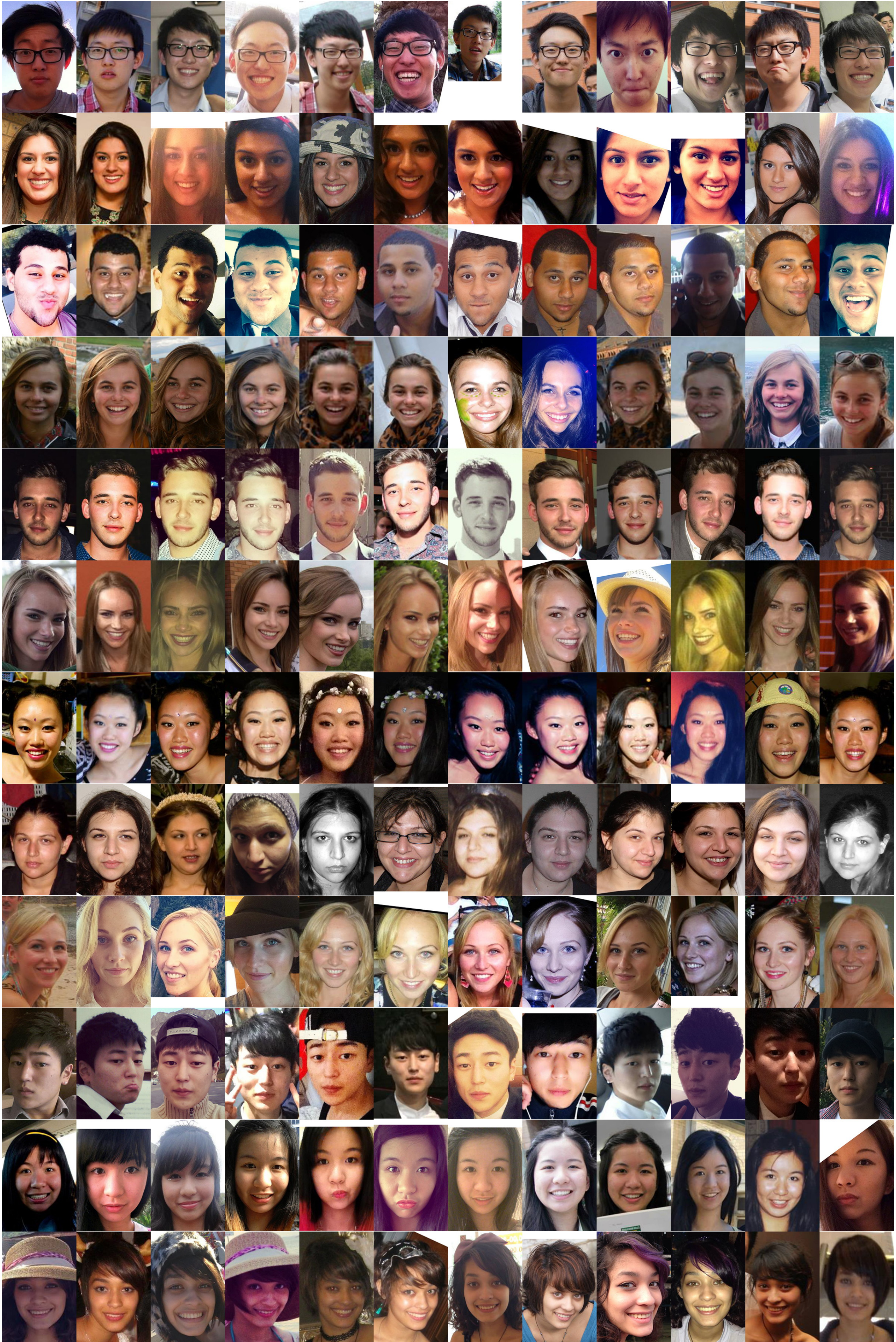

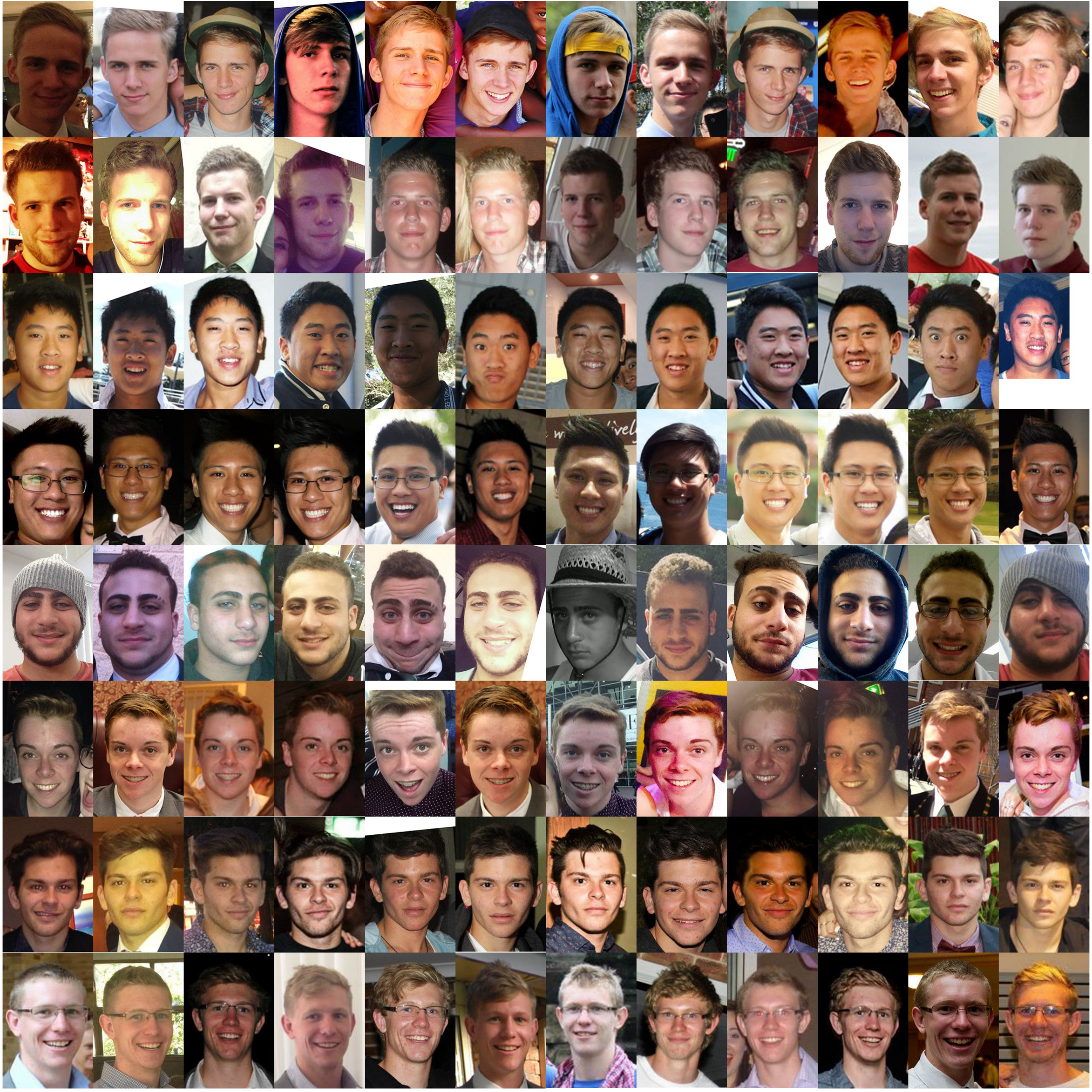

Supplement: Supplementary file 2 — Images used in the Calibration experiment. (PDF 16.7 MB) [file 41235_2017_58_MOESM2_ESM.pdf]
