# Supplementary material for: Choosing face: The curse of self in profile image selection
Source: Cogn Res Princ Implic. 2017 Apr 14;2:23. doi: 10.1186/s41235-017-0058-3 (PMC5391387; doi:10.1186/s41235-017-0058-3)

The curse of self in profile image selection

D. White[1], C. A. M. Sutherland[2], A. L. Burton[3]
[1]UNSW Australia; [2]University of Western Australia; [3]University of Sydney

Additional File 5: Stimuli used in *Selection Experiment*

Stimuli are presented in matrices separately for self and other selections, with different network contexts presented on separate pages.

To aid perceptual comparison of profile image selections, locations of identities in the matrix correspond across pages, and most/least likely selections are presented on consecutive pages.

Masked images are of identities that did not consent to their images being used for publication. To ensure anonymity of participant data, we do not provide participant numbers associated with images here.

A full version of the Profile Image Database, enabling mapping of images to item rating data are available for use in academic research by contacting the lead author:

david.white@unsw.edu.au / davidwhitephd@gmail.com

Professional network context

Self (least likely)

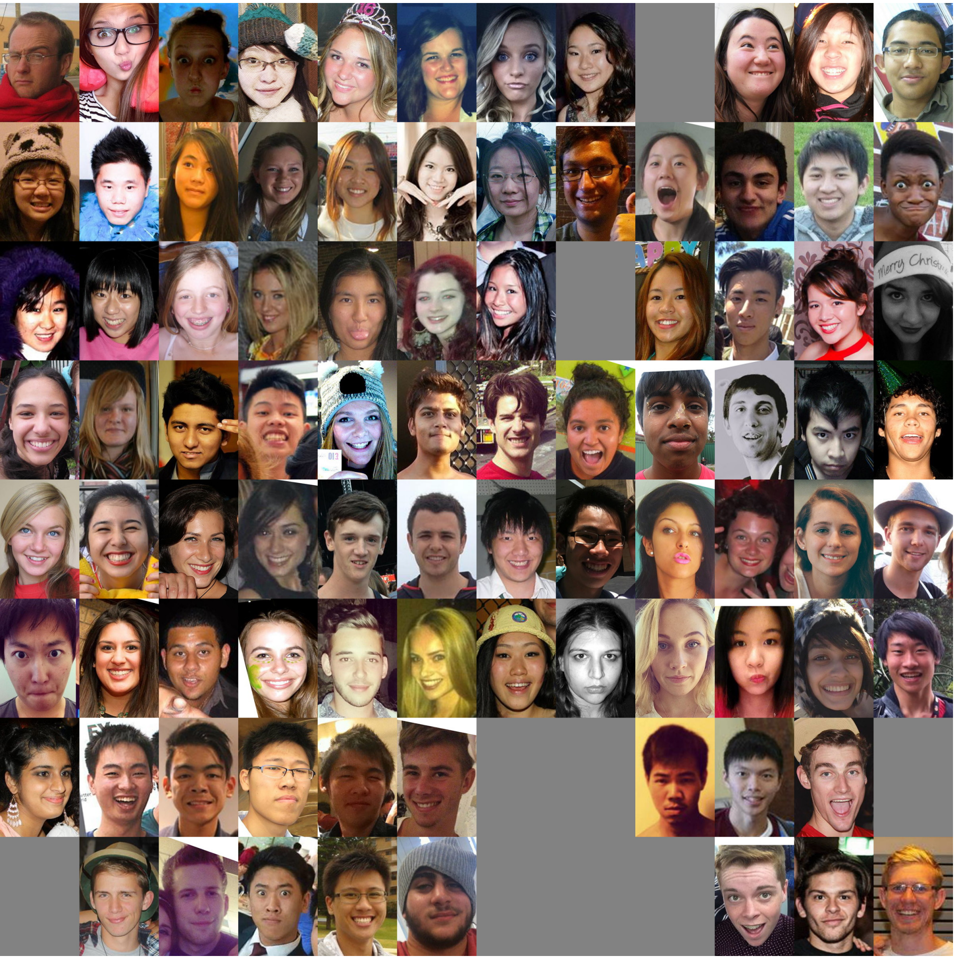

Other (least likely)

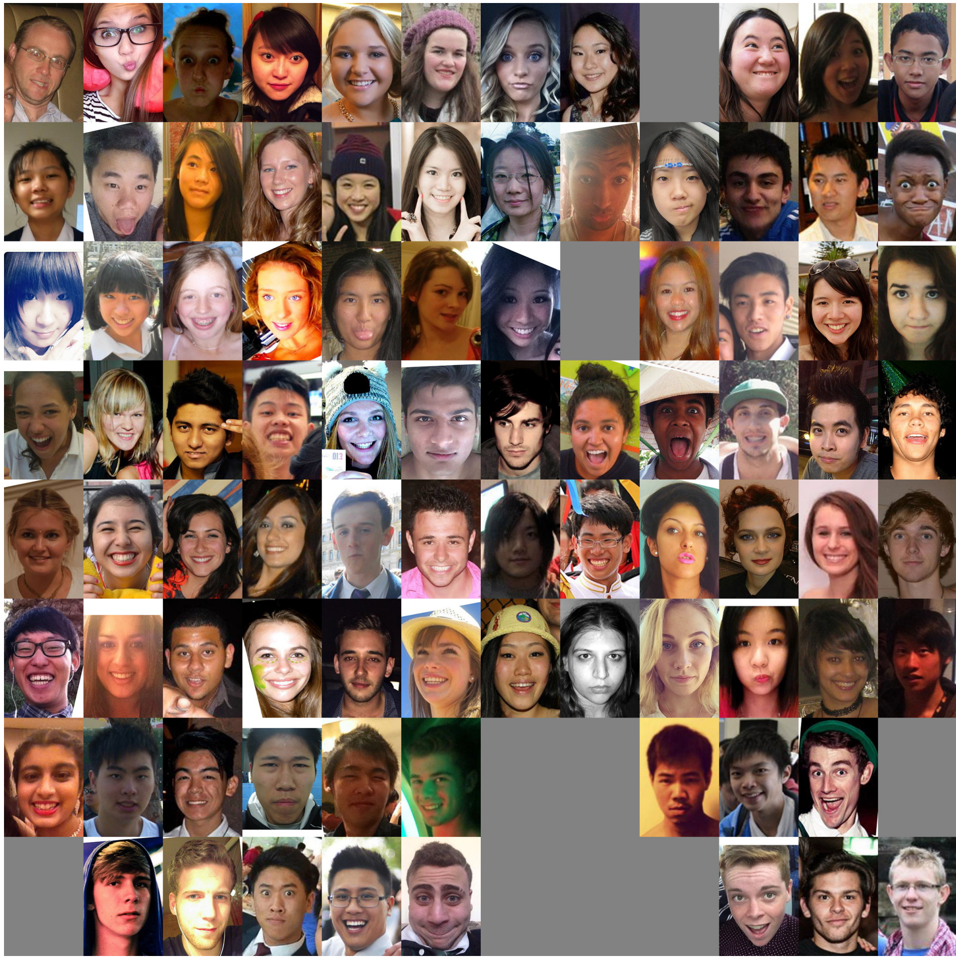

Professional network context

Self (most likely)

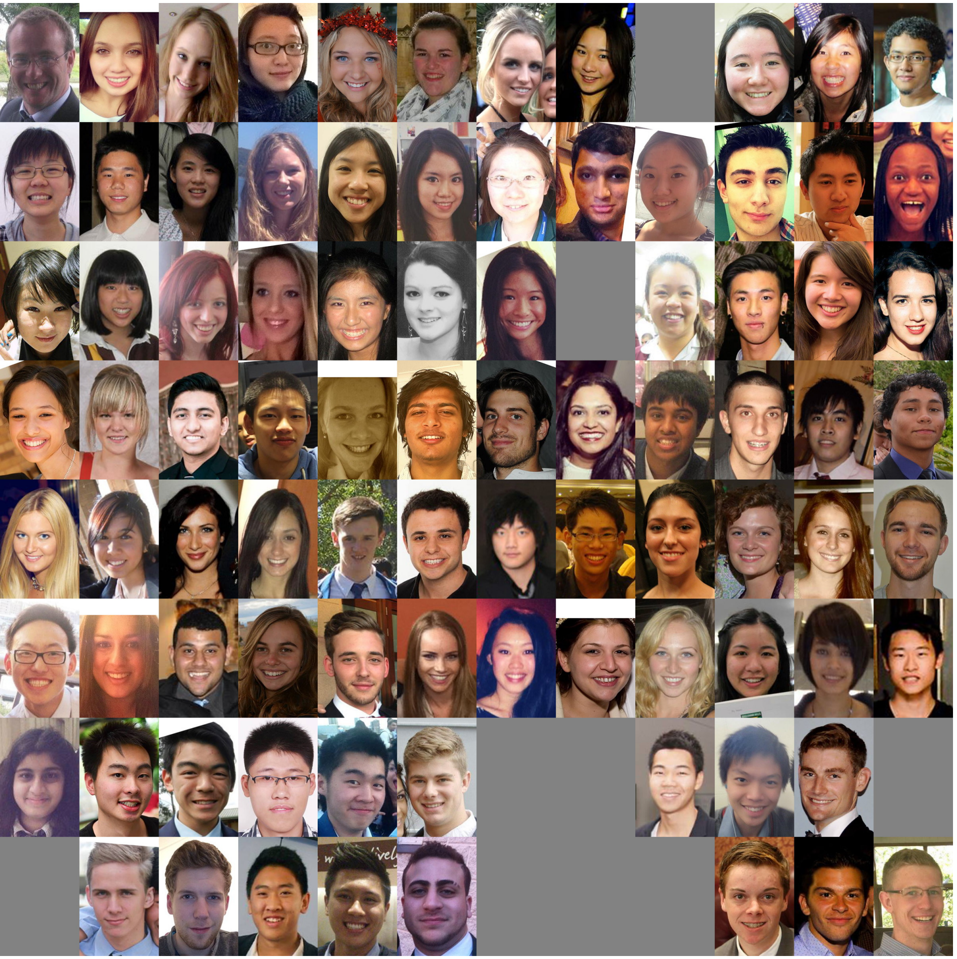

Other (most likely)

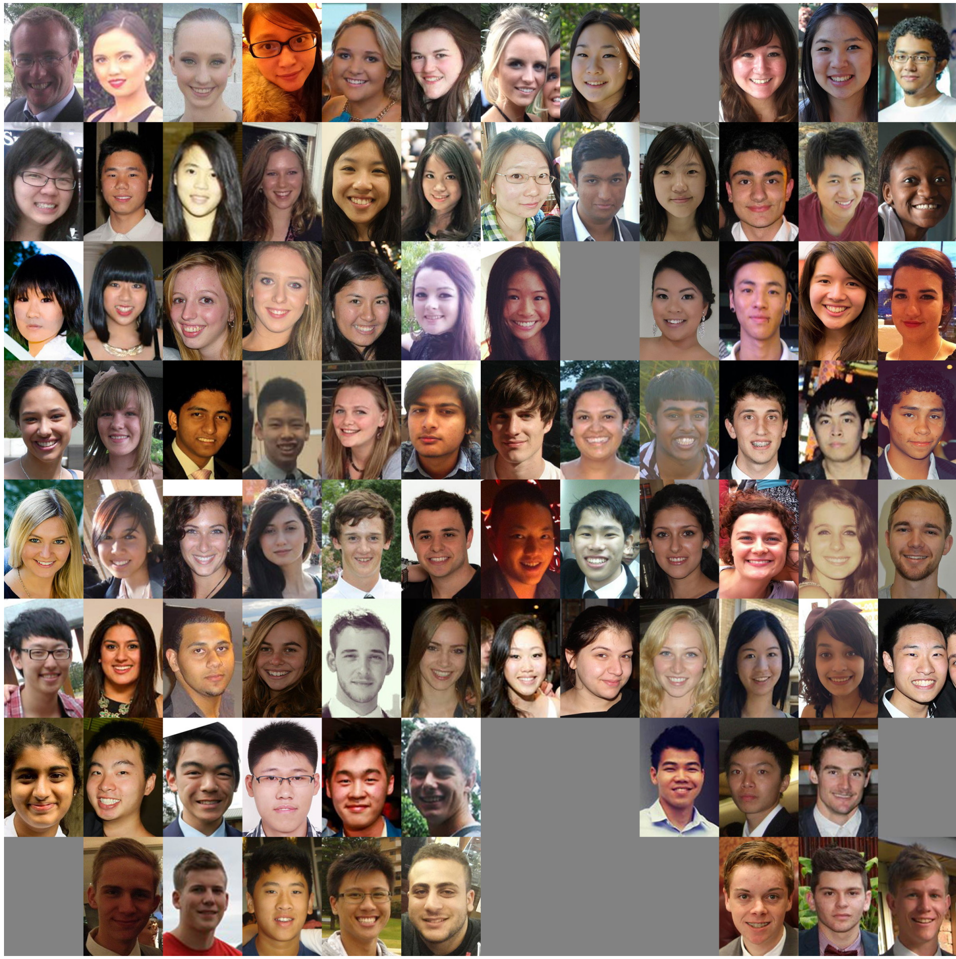

Dating context

Self (least likely)

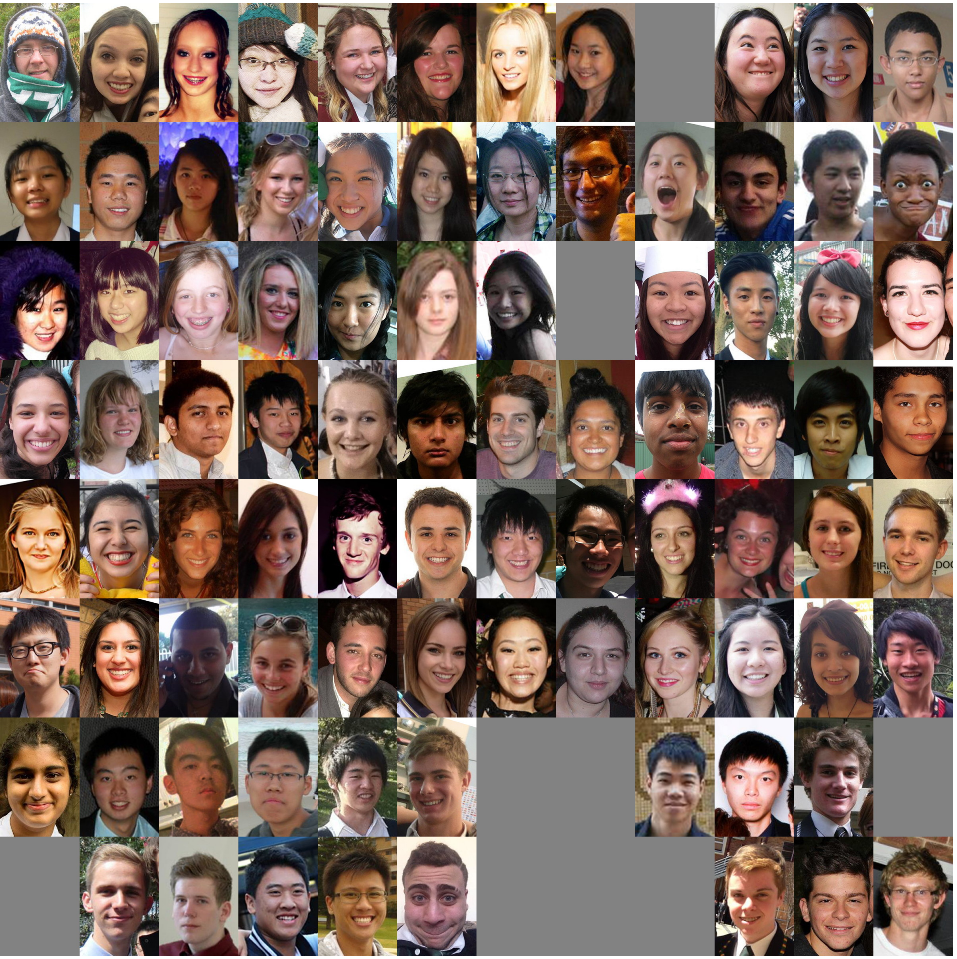

Other (least likely)

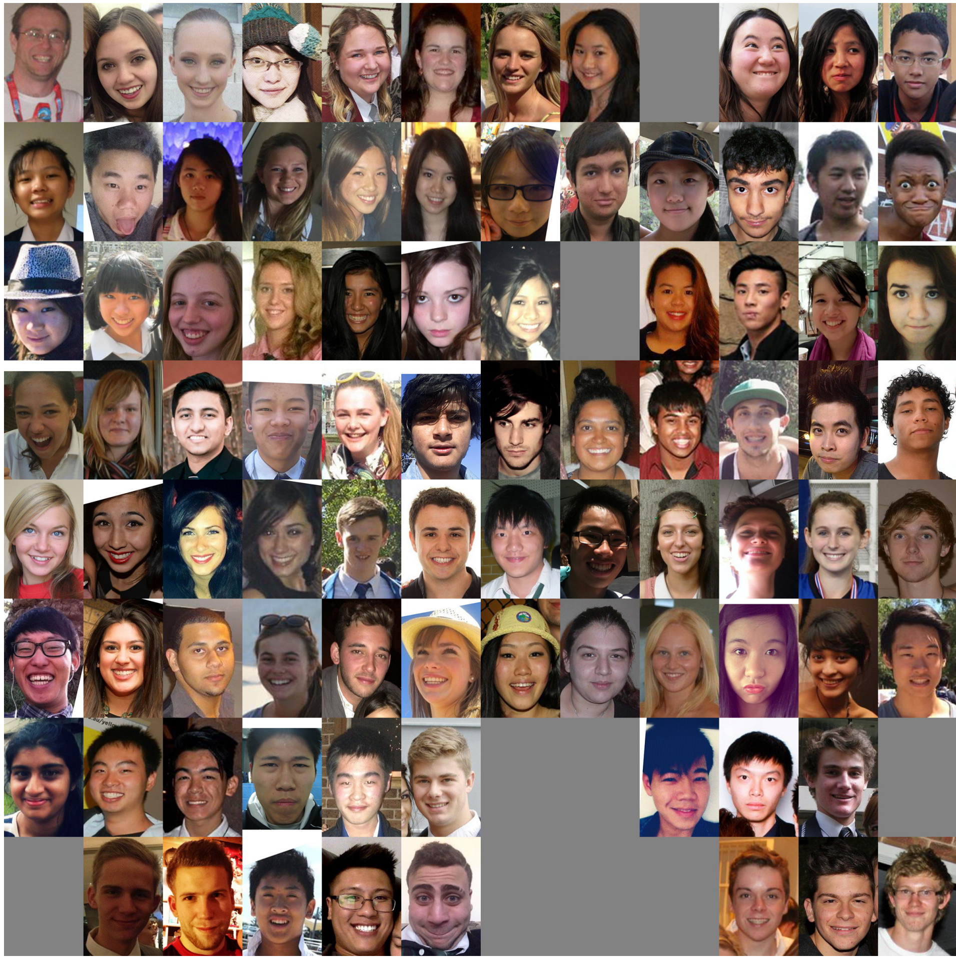

Dating context

Self (most likely)

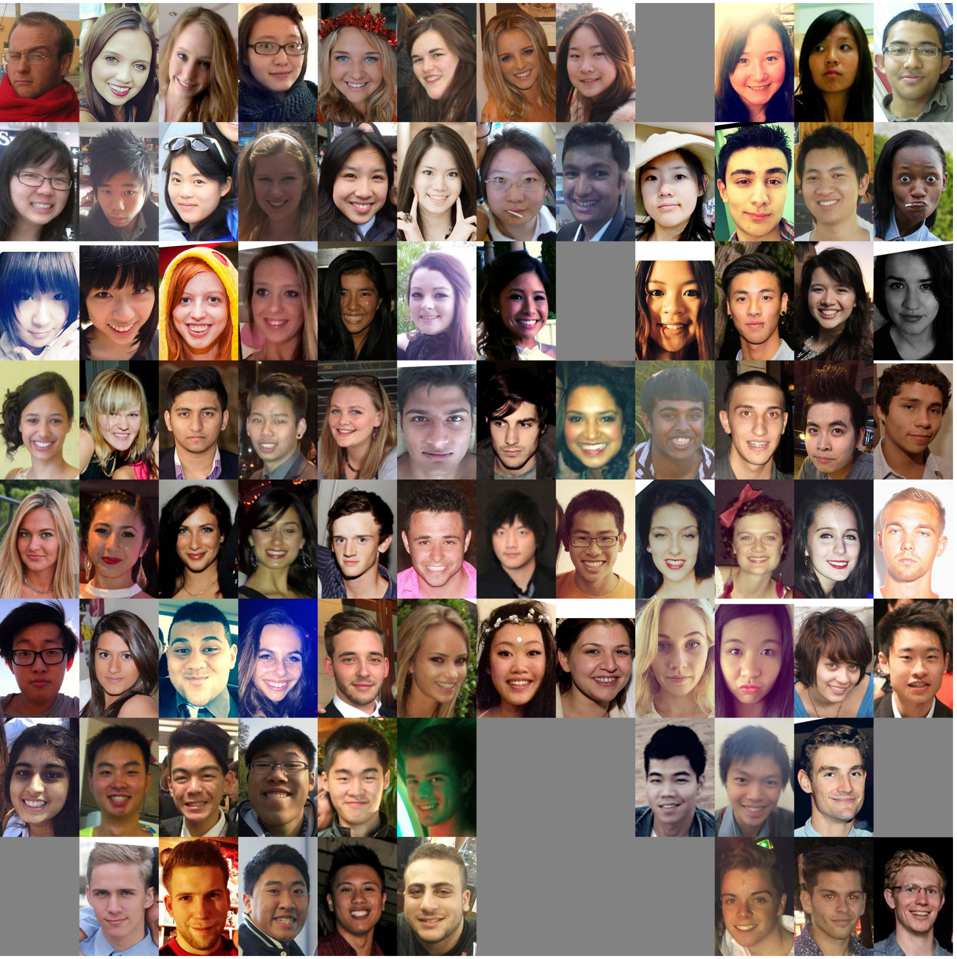

Other (most likely)

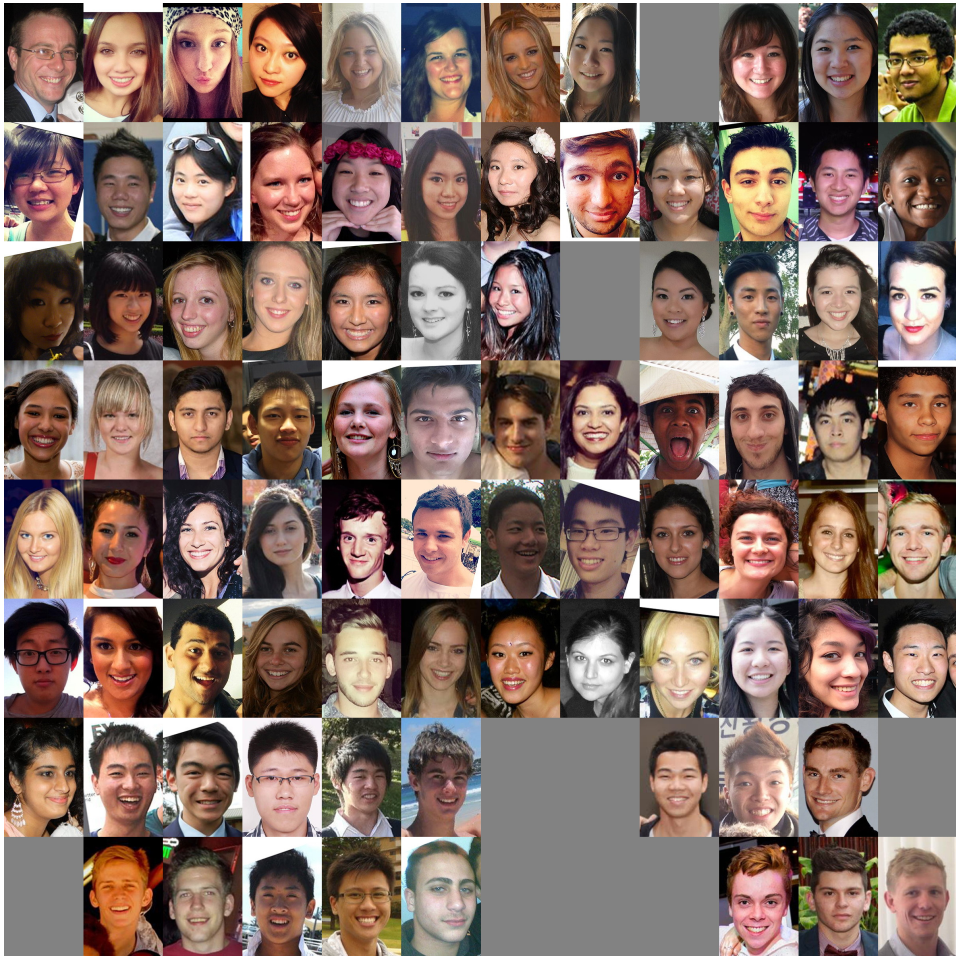

Facebook context

Self (least likely)

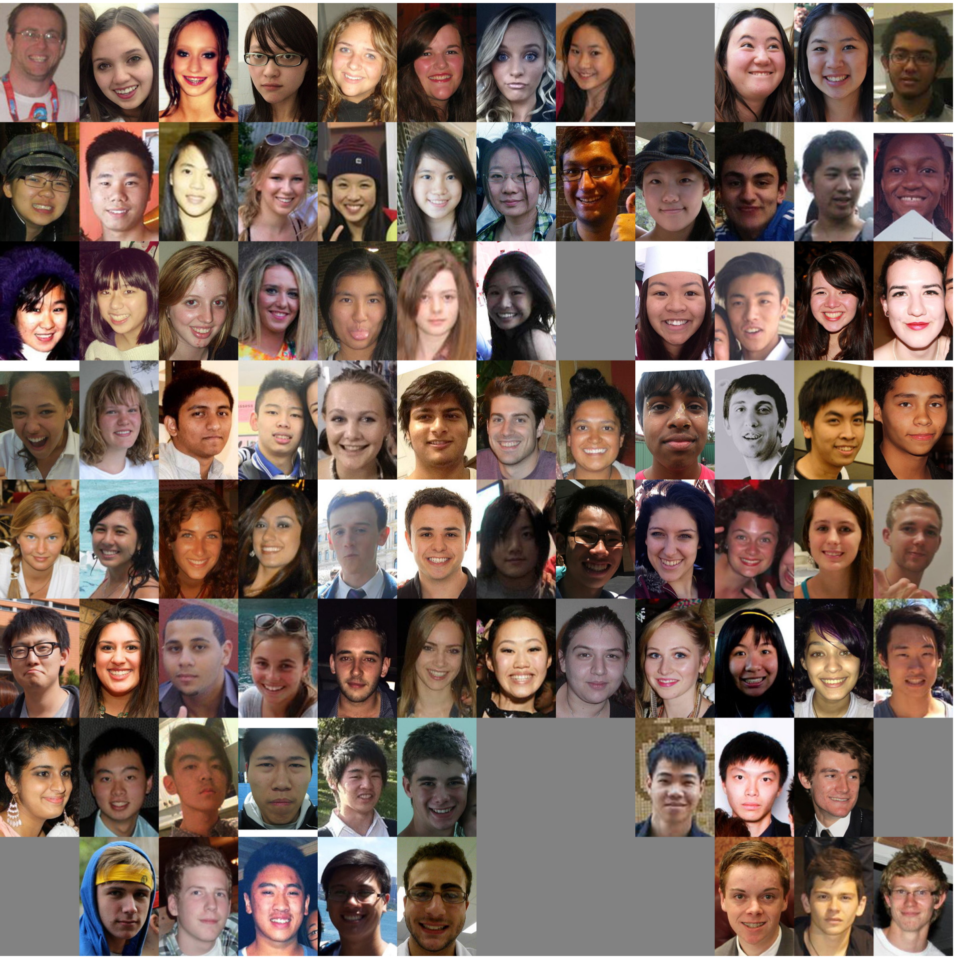

Other (least likely)

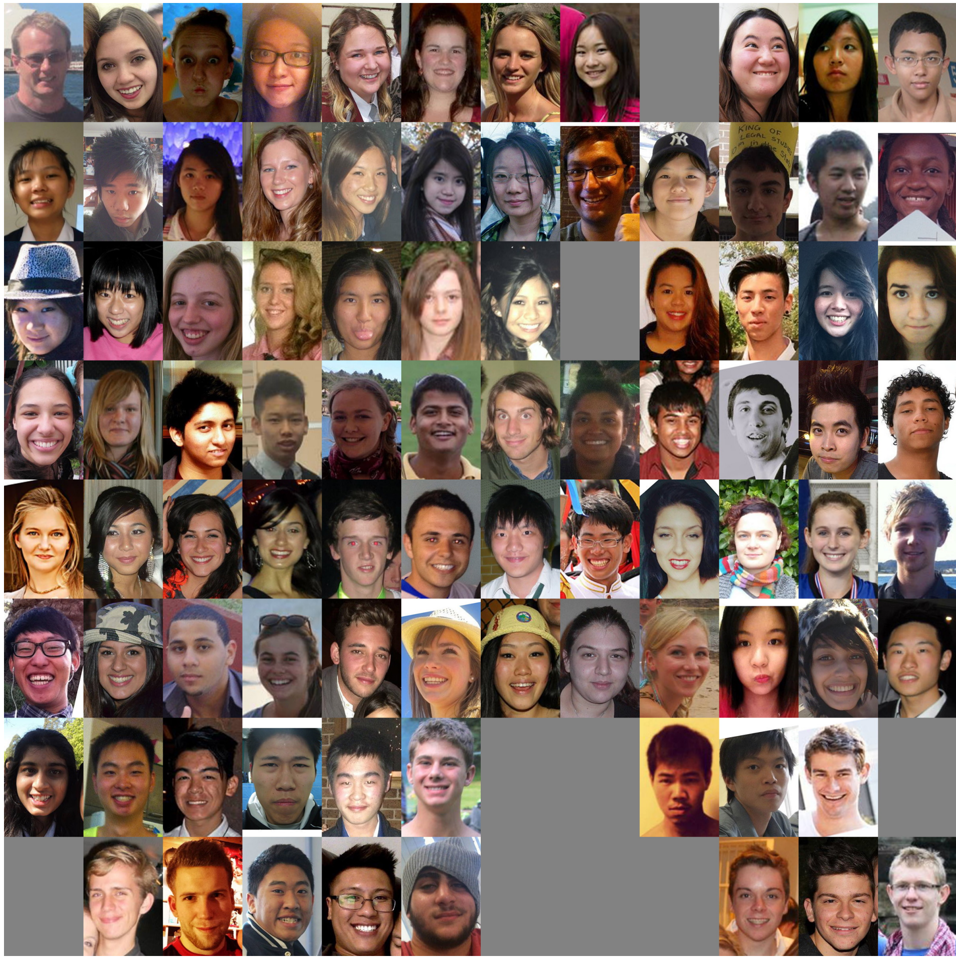

Facebook context

Self (most likely)

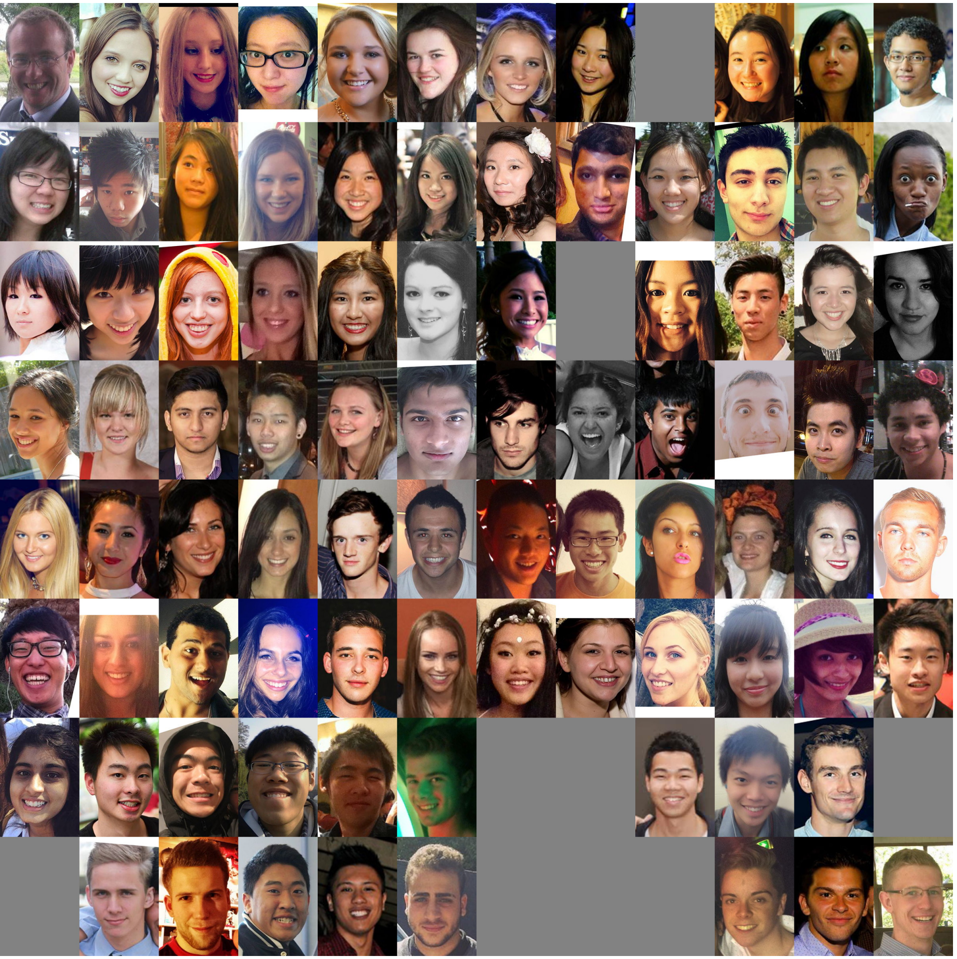

Other (most likely)

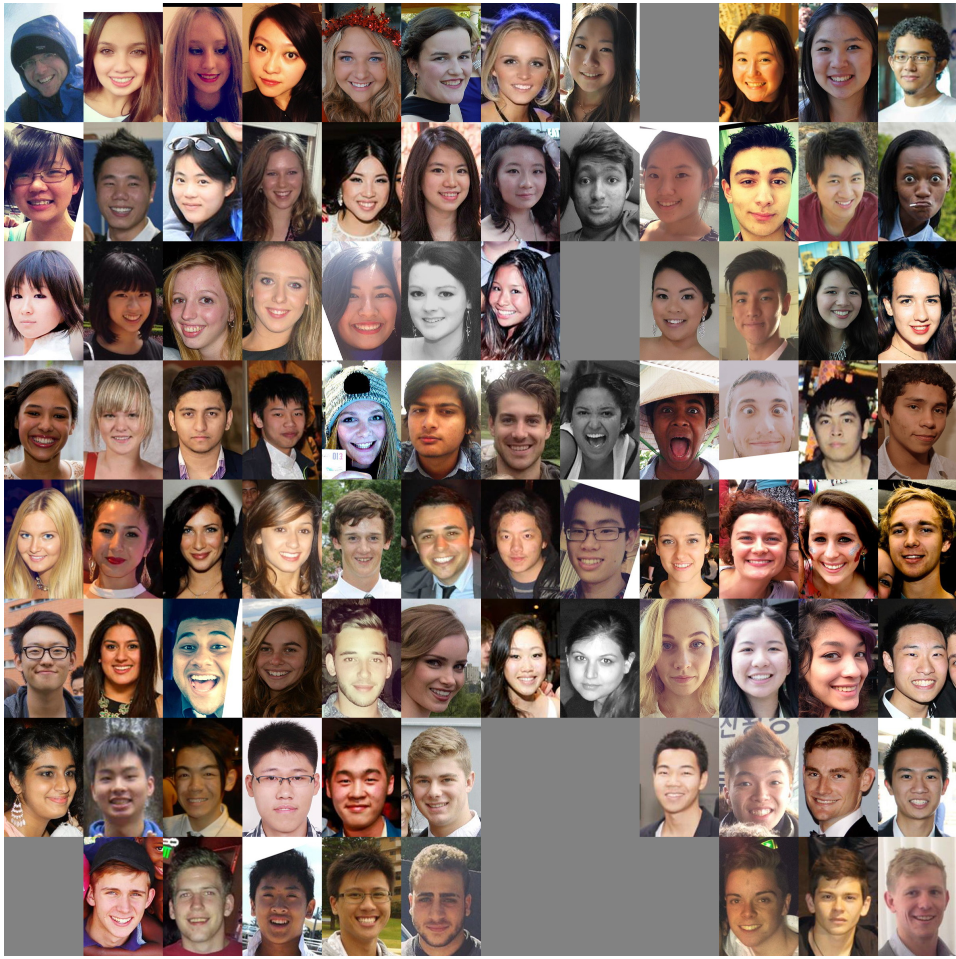

Supplement: Supplementary file 5 — Images used in the Selection experiment. (PDF 17.0 MB) [file 41235_2017_58_MOESM5_ESM.pdf]
