# Supplementary material for: Correction to: Choosing face: The curse of self in profile image selection
Source: Cogn Res Princ Implic. 2021 Aug 13;6:55. doi: 10.1186/s41235-021-00320-2 (PMC8363730; doi:10.1186/s41235-021-00320-2)
Supplement: Supplementary file 1 — Additional file 1. Full description of analysis in the Calibration experiment. [file 41235_2021_320_MOESM1_ESM.docx]

**The curse of self in profile image selection**

**Supplementary Materials (S4): Calibration Experiment Analysis**

David White*, Clare A. M. Sutherland, Amy L. Burton

*^*^Corresponding author: david.white@unsw.edu.au*

Calibration data were computed as Spearman’s Rho coefficients between selection likelihood ratings (Facebook, dating, professional) and trait ratings (attractiveness, dominance, trustworthiness, competence, confidence). These calibration scores were computed between the likelihood ratings and selectors own trait ratings, and also between likelihood ratings and trait ratings by a group of unfamiliar viewers recruited via the Internet. These data are summarized in Figure 2, and provided in full in Supplementary Materials (S3). Calibration scores were analysed by separate three-way mixed factor ANOVA with between-subject factor of Selection Type (self, other) and within-subject factors Context (Facebook, dating, professional) and Trait (attractiveness, dominance, trustworthiness, competence, confidence).

***Own ratings***

For own ratings, the main effect of Selection Type was non-significant, F (1, 202) = 1.48, p = 0.225, η_p_^2^ = 0.007. Main effects of Trait, F (4, 808) = 45.5, p = 0.003, η_p_^2^ = 0.184, and Context, F (2, 404) = 22.3, p = 0.011, η_p_^2^ = 0.099, were highly significant. There was a significant two-way interaction between Trait and Selection Type, F (4, 808) = 13.9, p < 0.000, η_p_^2^ = 0.065. Analysis of Simple Main Effects revealed that this interaction was driven by: (i) significantly lower calibration of self-selection with trustworthiness ratings, F (1, 202) = 9.75, p = 0.002, and competence ratings, F (1, 202) = 5.49, p = 0.020, compared to other-selection calibrations; (ii) *higher* calibration of self-selection with selector’s own dominance ratings, F (1, 202) = 10.6, p = 0.001; (iii) a non-significant difference between self and other selection calibration for attractiveness ratings, F (1, 202) = 0.68, p = 0.411.

The interaction between Context and Selection Type, F (2, 404) = 4.16, p = 0.016, η_p_^2^ = 0.020, was also significant, reflective of a higher calibration between selection likelihood and selectors own trait ratings for other-selections in professional, F (1, 202) = 5.73, p = 0.018, η_p_^2^ = 0.028, but not Facebook, F (1, 202) = 0.413, p = 0. 521, η_p_^2^ = 0.002, or dating contexts, F (1, 202) = 0.035, p = 0.852, η_p_^2^ < 0.000.

A significant two-way interaction between Trait and Context was also observed, F (8, 1616) = 22.3, p = 0.011, η_p_^2^ = 0.099, but this was qualified by a significant three-way interaction, F (8, 1616) = 3.73, p < 0.000, η_p_^2^ = 0.018. In light of the three-way interaction, we analysed Trait and Context interaction separately for self and other selection. Calibration of self-selection to Trait varied as a function of selection Context for attractiveness, F (2, 1010) = 54.9, p < 0.001, η_p_^2^ = 0.10, dominance, F (2, 1010) = 9.32, p < 0.001, η_p_^2^ = 0.02, and confidence, F (2, 1010) = 7.89, p < 0.001, η_p_^2^ = 0.02 (with non-significant main effects of trustworthiness, F (2, 1010) = 1.06, p > 0.05, and competence F (2, 1010) = 0.90, p > 0.05). For other-selection, calibration between varied as a function of selection Context only for attractiveness ratings, F (2, 1010) = 55.1, p < 0.001, η_p_^2^ = 0.10. (Non-significant simple main effects of trustworthiness: F (2, 1010) = 0.57, p > 0.05; dominance: F (2, 1010) = 2.90, p > 0.05; competence: F(2, 1010) = 2.89, p >0.05; confidence: F (2, 1010) = 1.52, p > 0.05).

***Internet ratings***

For Internet ratings, the main effect of Selection Type was significant, F (1, 202) = 5.50, p = 0.020, η_p_^2^ = 0.026. Main effects of Trait, F (4, 808) = 8.77, p < 0.000, η_p_^2^ = 0.042, and Context, F (2, 404) = 5.25, p = 0.006, η_p_^2^ = 0.025, were also significant.

The only significant interaction was between Trait and Context, F (8, 1616) = 28.3, p < 0.000, η_p_^2^ = 0.123. This reflected greater calibration between selection likelihood and trait judgments where traits aligned to network contexts, for example there was higher calibration for attractiveness in dating relative to other traits (Simple Main Effect of Context on Attractiveness: F (2, 406) = 6.17, p = 0.002, η_p_^2^ = 0.099) and for competence in professional networks relative to other traits (Simple Main Effect of Context on Competence: F (2, 406) = 31.5, p < 0.000, η_p_^2^ = 0.135).

We found non-significant interactions between Context and Selection Type, F (2, 404) = 0.297, p = 0.743, η_p_^2^ = 0.001, Trait and Selection Type, F (4,808) = 1.847, p = 0.118, η_p_^2^ = 0.009, and a non-significant three-way interaction, F (8, 1616) = 0.580, p =0.648, η_p_^2^ = 0.004.
